# Anisotropic Electrostatics in the Instability of GLP‑1 Analog Micelles: Effects of Electrolytes, Denaturants, pH, and Temperature

**DOI:** 10.1021/acsomega.6c02639

**Published:** 2026-08-10

**Authors:** Curtis W. Jarand, Ivan Zemskov, David Müller, Andreas Stadelmaier, Laurin Melzig, Ralph Schönleber, Wayne F. Reed

**Affiliations:** † 5783 Tulane University, New Orleans, Louisiana 70118, United States; ‡ 200740 Bachem AG, 4416 Bubendorf, Switzerland

## Abstract

Glucagon-like peptide-1 (GLP-1) analogs (GLPA) exist primarily as micelle-like associations when free in aqueous solution. The results here indicate that anisotropic electrostatic interactions play a central role in the instability and aggregation, which appear to arise predominantly from multipole, orientation-dependent electrostatics: net dipole moment and charge in GLPA affect attraction and repulsion, features not captured by mean-field, spherically symmetric approaches. Whereas hydrophobicity drives the micelle formation, electrolyte-dependent aggregation appears to be governed by these electrostatic interactions. Increasing ionic strength screens the Coulomb repulsion between micelles, reducing the electrostatic stabilization barrier and allowing orientation-dependent multipole attractions to promote aggregation. This behavior contrasts with globular protein aggregation, typically dominated by the classical hydrophobic effect. Spectroscopically monitoring forward and reverse dialysis with a custom device, stability of liraglutide and semaglutide samples was mapped vs electrolyte (NaCl) and denaturant concentrations (guanidinium chloride, Gdn). Gdn^+^ cation binding to negatively charged amino acids reduces net charge and dramatically destabilizes GLPA. In contrast, simple cations, such as Na^+^, merely screen electrostatically, and no binding term is required to explain the data. Aggregation caused by both NaCl and Gdn^+^ was semi-irreversible. An electrostatic model, based on attractive, screened monopole-dipole, dipole–dipole, and repulsive monopole-monopole interactions was developed to interpret results. This model may be applicable to other peptides and biologics with asymmetric and patchy charge distributions, and dipole moments. The work establishes a stability-testing paradigm that may accelerate development of these biologics, as well as other therapeutic peptides.

## Graphical abstract

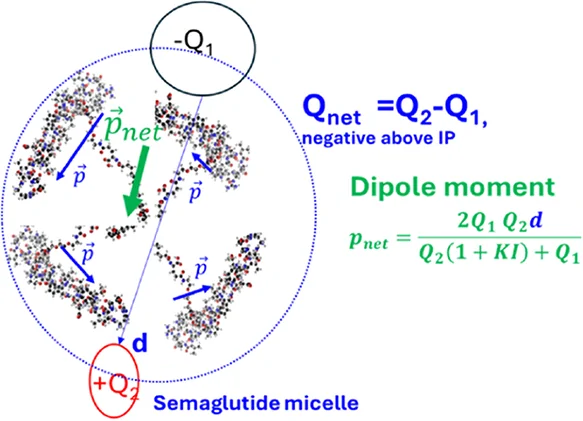

## Introduction

Glucagon-like peptide1 (GLP-1) is an incretin, a hormone derived from the gut and released from enteroendocrine L-cells after food intake, distributed along the entire gastrointestinal tract, leading to the secretion of insulin from pancreatic β cells in a nutrient-dependent manner.^1^ GLP-1 is also secreted from α cells in the pancreas and multiple other regions in the central nervous system. There are two known bioactive forms of this peptide hormone in the bloodstream: GLP-1- ^7–36^ -NH_2_, which is with ca. 80% the more abundant form, and GLP-1-,^7–37^ the less abundant form with roughly 20%. GLP-1 has a relatively short half-life of approximately 2–5 min once released into plasma. This is partly a result of the action of the enzyme dipeptidyl-peptidase-4 and GLP-1′s low molecular weight, which accelerates its renal clearance.^2,3^

The extensive anatomical distribution and range of physiological roles of GLP-1 suggest its important pharmacotherapeutic potential. GLP-1 receptor agonist therapies have been used mainly to treat type 2 diabetes mellitus and obesity.^4^ More recently, new medications, e.g., semaglutide, have become popular because of their efficacy in reducing body weight in obese patients with and without diabetes.^5,6^ At present, there are eight GLP-1 receptor agonists approved as medications for type 2 diabetes, obesity and cardiovascular disease: Albiglutide, dulaglutide, exenatide, extended-release exenatide, liraglutide, lixisenatide, semaglutide and tirzepatide and many more are undergoing clinical development.^7^

In comparison to the extensive pharmacological characterization of GLP-1 analogs (GLPA), the physical chemistry of GLPA solutions is not as well understood, and the fundamental physical origins of their instability have not been quantified.

GLPA form oligomeric, micelle-like species due to self-assembly. The self-association is primarily caused by their peptide nature and amphiphilic modifications. GLPA molecules in solution are prone to physical instabilities, particularly aggregation. Physical aggregation, driven by noncovalent interactions such as hydrophobic effects, electrostatic forces, and mechanical stress, can occur during manufacturing, handling, or storage. Understanding the physical aggregation behavior of this class of peptides is essential for ensuring product stability, efficacy, and safety.^8–11^

The propensity of liraglutide to form fibrils is strongly dependent on pH.^9,12^ Below pH 8.2, even small changes in pH result in a marked decrease in stability.^9^ Semaglutide at pH 7.4 is substantially less prone to fibril formation than liraglutide at pH 7.3–8.2; however, lowering the pH to 6.7 also induces fibril formation by semaglutide.^9^ The anisotropic electrostatic model below explains why pH should have such a large effect, in that it significantly controls net charge and dipole moments.

The theoretical isoelectric points (pI) are approximately 4.9 for Liraglutide and 5.4 for Semaglutide. In addition, the pI of Semaglutide was experimentally determined at pH 5.01, at which zeta potential crossed zero. Around the pI in the approximately range of pH 4.0–6.5, larger particles were detected, indicating reduced solubility, whereas in the range of pH 3.1–4.0 and pH > 6.5 semaglutide was fully soluble. The GLPA sequences contain several ionizable residues, including Asp and multiple Glu residues, as well as terminal charge groups and the fatty-acid side chain. Under the alkaline conditions used in much of the present work, these acidic groups are expected to be largely deprotonated, contributing to the net negative charge and dipole moment considered in the electrostatic model.

The main goal of this work is to quantitatively assess the instability of GLPA, manifested as aggregation, against ionic strength (NaCl in this case), denaturant (guanidine-HCl in this case), and temperature stressors, and to build a corresponding model to explain the origins of instability. The use of the cuvette-based dialysis method allows the response of the GLPA to a continuous concentration change of electrolyte and denaturant to be monitored. A qualitative overview of these effects, together with considerations of impurities in GLPA has recently appeared,^13^ while another review covers stability of GLPA under pH, temperature and ionic strength,^14^ and in the presence of other oligopeptides.^15^ Several recent works examine instability of GLPA leading to colloidal aggregation.^16,17^

The current work investigates instability, not morphology, and the electrostatic model developed does not assume spherical symmetry, but rather explores whether orientation-dependent dipole interactions can account for observed aggregation behavior. Reducing the model to pointlike dipoles and net charges renders the model agnostic to the morphologies of GLPA monomers, micelles, and aggregates. In addition to interpreting the data trends, the model yields estimates of GLPA dipole moments, and fractional binding of the Gdn+ ion to negative amino acids, and associated binding constants. Of course, finer grained models will take into account the additional effects of morphology on GLPA stability. Effects of mechanical stressors should be explored in subsequent work.

Molecular simulations have shown that anisotropic surface charge distributions can create orientation-dependent electrostatic attractions between colloids.^18^ The work here treats these effects using monopole–dipole and dipole–dipole interactions. Data show the predicted salt-driven aggregation thresholds observed in GLP-1 analogs. Furthermore, the role of anisotropic electrostatics–as opposed to isotropic, mean field, net-charge based models—has experimental and theoretical support: Variations in net charge and dipole moment can strongly influence protein interactions.^19^ Permanent and induced dipoles have been shown to cause attractions between generic colloids with the same net charge.^20^ Orientation-dependent anisotropic electrostatics for heterogeneous, patchy charge distributions have been shown to be a vital extension of the simpler isotropic models.^21^ Orientation-selective binding has been shown to be tunable by modulating ionic strength and pH.^22^ It was demonstrated, explicitly for proteins, that patchy, anisotropic charge distributions strongly influence aggregation and crystallization.^23^

## Materials

R-&D samples of lyophilized semaglutide (SG) and liraglutide powders (LG) in salt form were donated by Bachem AG. All samples were shipped in the form of a white powder. SG/LG had the following characteristics: Purity,% (RP), 95.7/95.5 Purity, % (SEC), 99.9/99.9 Water content, %, 5.7/5.0. All materials (SG, LG1 and LG2) were used as sodium salts (SG: disodium, LG: trisodium).

Phosphate buffer for LG dissolution was prepared at 10 mM, pH 8.15 using monobasic sodium phosphate (≥99%) and dibasic sodium phosphate (≥99%), both obtained from Sigma-Aldrich. SG solutions were prepared in 18.2 mΩ ultrapure water. NaCl (ACS grade) and guanidine-HCl (98%) (Gdn) were obtained from Fisher Scientific. pH adjustment of solutions for studies at pH 6.7 and pH 10.0 was performed by titrating with aliquots of HCl or NaOH to the desired pH.

## Methods

### Spectroscopic Monitoring during Dialysis

The custom-built dialysis device inserts into a standard 1 cm cuvette, and can be used without modification on any instrument accepting a standard 1 cm cuvette. The device has been described elsewhere.^24^ The current, improved version incorporates a miniature (mm-scale) ion selective field effect transistor^25^ (ISFET, Sentron, Leek, Netherlands) and noncontact stir (“noncontact stir” means the impeller does not touch any part of the cuvette, unlike, for example, a magnetic stir bar). In summary, via a dialysis membrane, the device partitions the contents of the cuvette into Fluid 1, which contains the biologic or other substance of interest and which is interrogated by the spectroscopic instrument (SLS and DLS in this work), and Fluid 2, which contains the dialysate. The dialysate in Fluid 2 is continuously circulated through a closed external reservoir and flow path. The circulating dialysate can also be monitored externally with conductivity, pH, or optical sensors. A simplified representation is given in Scheme 1.

**Scheme 1:**
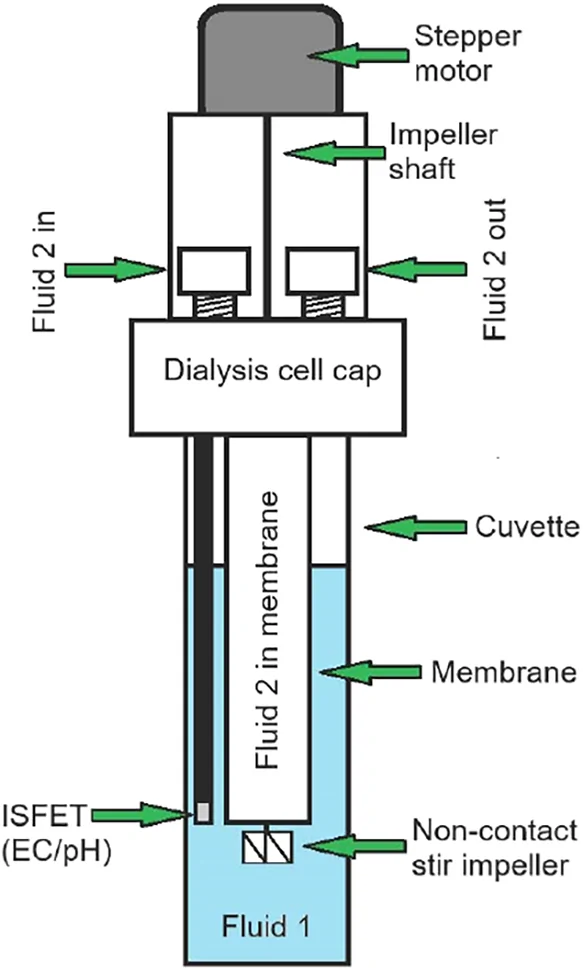
Simplified Drawing of the Spectroscopic Dialysis Device

Use of the dialysis device has several advantages: It uses a single sample, which sweeps through a wide range of excipient concentrations in a single run, giving more accurate and precise results, using less sample, and being far less labor-intensive than mixing individual samples. For those methods that use robots to prepare multiple discrete samples, this method eliminates the robots. Also, by mixing discrete samples, instabilities can be missed by lag times between sample preparation and measurements. In contrast, since solution behavior is monitored in real-time as it changes, there is no lag time between a solution condition and the measurement of its immediate effects. A further advantage is the ability to recover sample after dialysis. Because the cuvette remains stationary within the light scattering or other spectroscopic instrument, there are no moving parts in the optical train, and no alignment, meniscus, shear, or edge effects that can occur in systems, such as well-plates, that move multiple samples with respect to the light source and detection apparatus.

Complementing the dialysis method were temperature ramps (T-ramps) in both SLS and DLS, as well as time-dependent monitoring of GLPA behavior under fixed concentrations of NaCl and Gdn, and at fixed T.

### Static and Dynamic Light Scattering (SLS and DLS)

The principal methods used were static and dynamic light scattering (SLS and DLS). DLS measurements were made on a Brookhaven Instruments Nanobrook Omni (Holtsville, New York) BI-90, with λ_0_ = 660 nm and on a BI90-Plus, with λ_0_ = 660 nm. The SLS measurements were carried out on an 18-sample cell Argen (Yokogawa Fluence Analytics, Houston, Texas), with λ_0_ = 660 nm. When an Argen detector saturates, the incident laser intensity is automatically reduced by one of several neutral density filters with optical densities 0.5, 1, 2.

SLS sampling was every five seconds. In contrast, in order to avoid a velocity-dependent term in the autocorrelation function of the stirred sample using DLS, a relay was used for stop-flow, so that stirring was stopped during the DLS measurement, and stirred by the integral noncontact stir impeller between measurements. This gave a two-minute cycle period between measurements. Early phenomena during dialysis, such as abrupt peaks in scattering were often missed by these DLS measurements, but captured by SLS.

In DLS the apparent hydrodynamic radius *R*_H,ap_ from the z-average self-diffusion coefficient, ⟨*D*_0_⟩_z_, and polydispersity index, PI, were obtained by standard second order cumulant analysis of the light scattering electric field autocorrelation function, g_1_(τ), derived from the measured intensity autocorrelation function g_2_(τ).

⟨*D*_0_⟩_z_ (found at low concentration, or by extrapolation to zero concentration) from DLS is used to compute the apparent z-average hydrodynamic radius *R*_H,ap_, starting with the Stokes–Einstein equation for monodisperse spheres of hydrodynamic radius *R*_H_ and self-diffusion coefficient *D*_0_

1 $$D_{0}=\frac{k_{B}T}{6\pi \eta R_{H}}$$

where *k*_B_ is Boltzmann’s constant, and η the solution viscosity. The dependence of η on *T* is corrected using readily available physical data from NIST or CRC sources on aqueous NaCl and Gdn solutions.

For polydisperse solutions ⟨*D*_0_⟩_z_ and *R*_H,ap_ are reciprocally related. Hence *R*_H,ap_ is actually the reciprocal of the *z*-average inverse *R*_H_, which can differ considerably from the true *z*-average hydrodynamic radius ⟨*R*_*H*_⟩_*z*_

2 $$R_{H,\mathrm{ap}}=1/{\left\langle 1/R_{H}\right\rangle }_{z}$$

The poly_d_ispersity index, PI, from the DLS autocorrelation function is the ratio of the second moment to the first moment squared of the cumulant expansion of the logarithm of the electric field autocorrelation function

3 $$\mathrm{PI}=\frac{{\left\langle D_{0}^{2}\right\rangle }_{z}-{\left\langle D_{0}\right\rangle }_{z}^{2}}{{{\left\langle D_{0}\right\rangle }_{z}}^{2}}$$

PI < 0.10 is often considered low polydispersity, 0.10 < PI ≤ 0.25 medium polydispersity, and PI > 0.25 high polydispersity.

The single angle DLS and SLS do not provide any direct information on the molar mass distribution or size distribution. Although most DLS software has built-in Laplace transform procedures for extracting particle size distributions, they deal with an inherently ill-conditioned transform problem, and these results are largely considered only qualitative, and not quantitatively reliable. For this reason, DLS results here are given only in terms of *R*_H,ap_ and PI. In viewing Laplace transforms of the autocorrelation functions of the data below, there is frequently a trimodal structure, corresponding roughly to micelles (<10 nm), the majority of the aggregates (∼100 nm) and a very small amount of large scatterer (∼1000 nm), perhaps “dust” or very large aggregates. There is not enough quantitative certainty in these distributions for further analysis, so data are given below in terms of *R*_H,ap_, and PI from the cumulant analysis.

Furthermore, the single angle DLS and SLS data provide no morphological information on the GLPA.^26–29^ For example, there is evidence that GLPA contain a certain amount of α–helical structure,^10^ and β-sheets.^30^ The SLS and DLS methods are, however, exquisitely sensitive to changes in molar mass due to aggregation and dissociation, which is the focus of this work. Additionally, the pointlike multipole electrostatic model does not require morphological information.

### Monitoring at Fixed [NaCl], [Gdn], and T for Time Dependent Processes

All methods involving the ramp of some physical parameter (e.g., temperature, pressure, concentration of a molecule, etc.) can convolve the time scale of the ramp with the time scale of a sample’s physical change, if the two time scales are on the same order of magnitude. For the changes in the physical characteristics to be meaningful as a function of the ramp parameter, the sample’s time-dependent processes must be much faster than the ramp rate, so that the system is instantaneously in equilibrium at each point during the temporal ramp. Accordingly, when there is evidence of time-dependent effects on the time scale of the dialysis, complementary time-dependent measurements were made at fixed [NaCl], [Gdn], and T. This is especially important in the vicinity of sharp aggregation thresholds that were encountered in many of the dialysis experiments.

## Results

### Equilibrium Characterization of GLPA

Equilibrium SLS data show that the GLPA are associated in units of 5–8 GLPA monomers. The associations are due to the hydrophobicity introduced by the long alkyl chains on the GLPA. These will be termed ‘micelles’ throughout, even though they are different from surfactant and other micelles that have critical micelle concentrations. There have been several studies of the micellization and morphological properties of GLPA structures.^31,32^

The equilibrium weight average molar mass, *M*_w_, and second virial coefficient *A*_2_ were determined by static light scattering (SLS) at 90°. The size of the stable GLPA was around 5nm, which gives a negligible angular dependence for scattering with λ_0_ = 640 nm and 660 nm.

Figure 1 shows the data for SG, LG, used to compute *M*_w_ and *A*_2_. The values were determined by measuring scattering from discrete concentration aliquots, using the 10 mM pH 8.15 buffer for diluting successive aliquots. SG was measured in aqueous 100 mM NaCl, used to suppress polyelectrolyte scattering effects (decreased scattering at low ionic strength).

**Figure 1:**
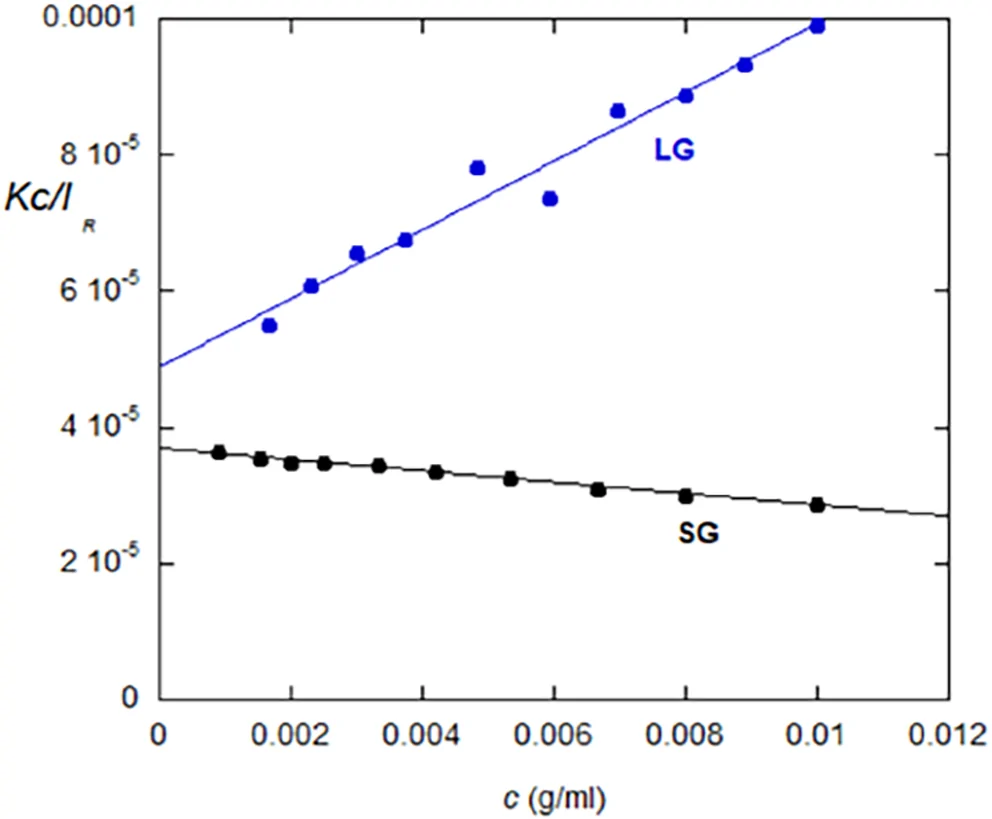
Determination of *M*_w_ and *A*_2_ at equilibrium for SG and LG. LG was diluted with 10 mM pH 8.15 buffer. SG was prepared in and diluted with aqueous 100 mM NaCl.

*M*_w_ and *A*_2_ were determined by the usual procedure of linear fitting to *K*_c_/*I*_R_ vs *c*, where *c*(g/cm^3^) is GLPA concentration and *I*_R_ is the measured Rayleigh Scattering Ratio (1/cm, and calibrated via toluene’s known Rayleigh Ratio), and the angle-independent expression is

4 $$\frac{K_{c}}{I_{R}}=\frac{1}{M_{w}}+2A_{2}c$$

where *K* is the optical constant defined for vertically polarized incident light by

5 $$K=\frac{4\pi^{2}n_{0}^{2}{\left(\partial n/\partial c\right)}^{2}}{\lambda_{0}^{4}N_{A}}$$

where *n*_0_ = 1.33 is the aqueous solvent index of refraction, λ_0_ = 660 nm (or 640 nm for the BI-90) is the vacuum wavelength of the incident laser beam, *N*_A_ is Avogadro’s number, and the differential index of refraction is $\frac{\partial n}{\partial c}=0.185\;{\mathrm{cm}}^{3}/g$ . *I*_R_ was found from raw light scattering voltage by reference to toluene scattering of *I*_R_ = 1.19 × 10^–5^ cm^–1^ at 660 nm, and *I*_R_ = 1.35 × 10^–5^ cm^–1^ at 640 nm, and *T* = 25 °C.

Table 1 shows the analyzed SLS results for SLS determinations from Figure 1. It is noted that the *A*_2_ term produces a fractional value of the *M*_*w*_ term in eq 4 equal to *2A*_2_ *M*_w_ *c*. At 0.00035 g/cm^3^, the usual concentration for dialysis and iso-electrolyte monitoring, this amounts to about a 4% underestimate of *M*_w_ for LG, and similarly for SG. Hence, corrections for *A*_2_ can be considered second order.

**Table 1:** Equilibrium Properties of GLPA Micelles

	LG	SG in 100 mM NaCl
*M*_w_,_monomer_ (g/mol)	3750	4115
*M*_w_,_micelle_ (g/mol)	20,400	27,200
*A*_*2*_ (cm^3^ mol/g^2^)	0.0025	–0.00042
# monomers in micelle	5–6	7–8

Because DLS does not provide reliable distribution data, it can be surmised that the scattering is dominated by the GLPA micelles, whose masses are listed in Table 1, with a likely subpopulation of monomers that contributes little to the scattering. The stability and aggregation phenomena monitored are chiefly for the micelles of the GLPA, which are driven to form because of the long, hydrophobic fatty acid chain covalently bound to the peptide backbone. These micelles act as the “basic unit” of GLPA aggregation. Attractive dipole–dipole and monopole-dipole interactions may also add to the stability of the micelles, but the hydrophobicity of the fatty acid chains remains the main driver of micellization.

The data for SG in aqueous 100 mM NaCl suggest there may be a mass action effect on the micelles; namely as they are diluted, they lose monomers. Taking *I*_r_/*K*_c_ as a measure of *M*_w_, going from 0.010 g/cm^3^ to zero concentration 7,800 g/mol of molar mass are released, very close to two monomers. Dialysis and other experiments were performed at 0.0030 g/cm^3^ or 0.0035 g/cm^3^, which is low enough that mass-action effects should not be significant, and the basic unit for unstable aggregation is taken here as the micelles. Alternatively, the SG behavior may just correspond to a negative A_2_, as shown in Table 1, whereby the intermicellar interaction energy integrated over all mutual orientations is net negative, and there is no loss of mass upon dilution.

### NaCl Dialysis and Iso-[NaCl]

The GLPA are very sensitive to ionic strength and show nonmonotonic aggregation behavior. Figure 2 shows *R*_H,ap_ and polydispersity, PI, vs time, for dialysis of LG against 2 M NaCl. *R*_H,ap_ starts around 5nm, seen in the circled data point, but then abruptly jumps to above 50 nm, increasing to 150 nm, before starting to fall back toward 50 nm, and then slowly climbing again. PI essentially follows the pattern of *R*_H,ap_, increasing initially and then falling in unison with *R*_H,ap_, before monotonically increasing. During reverse dialysis PI is plateaued, whereas there is a slight increase and then decrease in *R*_H,ap_. The existence of a maximum and minimum for *R*_H,ap_ vs [NaCl] is predicted by the electrostatic model, discussed below.

**Figure 2:**
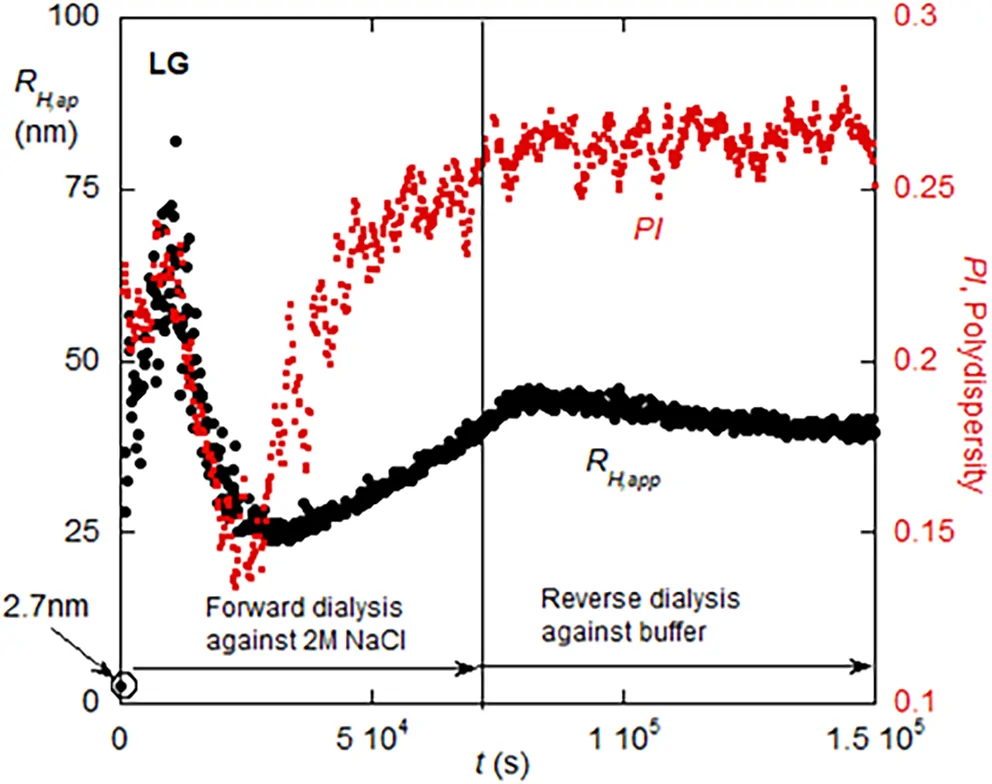
PI and *R*_H,ap_ vs time for LG forward dialysis against 2 M NaCl, and reverse. The LG was at pH 8.15 at a concentration of 0.010 g/cm^3^.

Figure 3a shows massive, transient colloidal aggregation of SG during dialysis against NaCl at pH = 6.7. *M*_w_/*M*_0_ is the normalized, dimensionless mass, where *M*_0_ is the initial weight average molar mass and *M*_w_ is the weight average mass at each instant. The detector hit saturation at about 700 mM, so the region from 700 mM to 840 mM is interpolated. Dialysis experiments under lower laser power showed the existence of the peak, but did not adequately capture the behavior prior to and after the peak. There is a linear increase in *M*_w_/*M*_0_ up until the threshold. After the peak *M*_w_/*M*_0_ decreases monotonically with an elbow at 1100 mM.

**Figure 3:**
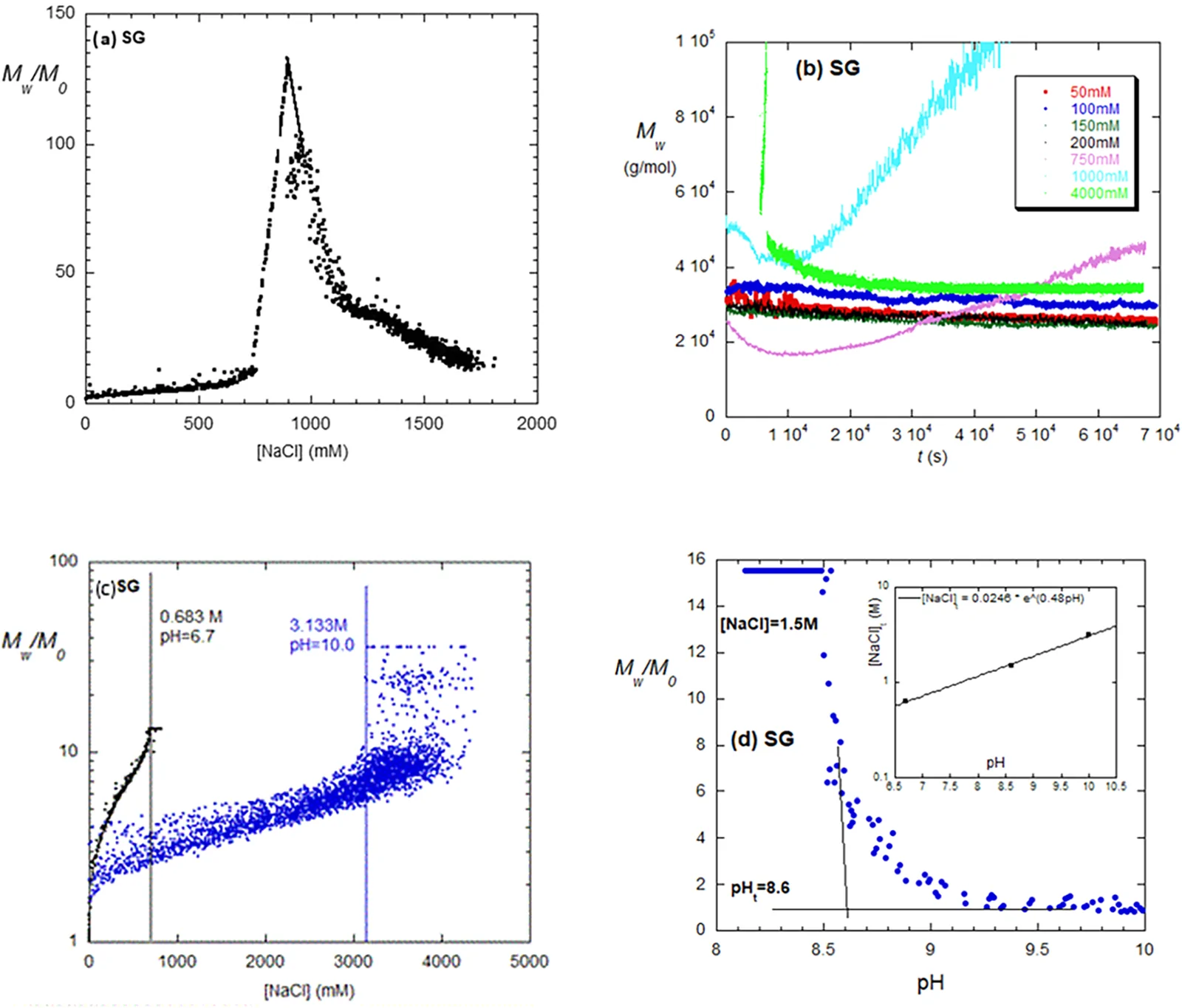
(a) *M*_w_/*M*_0_ for dialysis of SG at 0.0035 g/cm^3^ against NaCl at pH = 6.7. The detector abruptly saturated at 730 mM NaCl, so the values in the interval 730–840 mM NaCl are interpolated. (b) *M*_w_ vs *t* for SG at 0.0030 g/cm^3^ at discrete [NaCl]. (c) *M*_w_/*M*_0_ for SG dialysis against NaCl at pH = 6.7 and pH = 10, with SG concentration 0.0035 g/mL. The short horizontal bar at pH 6.7, and the dashed horizontal bar at pH 10 represent the saturation of the scattering photodetector. (d) 0.0035 g/cm^3^ SG at 1.5 M NaCl ramped from pH = 10 to 8.1. The pH threshold is pH_t_ = 8.6. The inset shows the threshold [NaCl]_t_ vs pH, with an exponential fit.

Figure 3b shows iso-[NaCl] data for discrete [NaCl]. This captures the stability of SG below the NaCl threshold for SG, [NaCl]_t_, the instability starting at 750 mM, continuing through 1000 mM up to 4000 mM. However, at 4000 mM the SG went through an immediate, massive aggregation followed by a rapid disaggregation to within range of the original SG micelles. The samples were prepared by having separate cuvettes with aqueous NaCl at the desired concentration. Then, an equal amount of stock semaglutide solution in buffer was introduced into each cuvette, the cuvette was gently mixed and then immediately introduced into the Argen for analysis. The delay between the SG being added to the solutions and the beginning of monitoring was approximately 5 s.

Figure 3c shows *M*_w_/*M*_0_ for the early portion of SG dialysis against NaCl at pH = 6.7 and pH = 10, with SG concentration 0.0035 g/cm^3^. The dialyses were stopped shortly after the threshold was reached. [NaCl]_t_ increases from 683 mM to 3133 mM going from pH 6.7 to 10.0. The higher threshold at pH = 10.0 is due to increased ionization of acid amino acids and loss of ionization of basic amino acids. This makes the net charge on the SG more negative, leading to higher repulsion, and the dipole moment lower, which provides weakened attractive potential. The net result is to require more NaCl to screen the higher net charge and to reach the negative dipole potential. This is covered in the electrostatic model, below.

Another means of finding the threshold is to fix [NaCl] and ramp pH downward starting from 10.0, until the threshold is reached. The measurements were made with the Sentron pH probe directly in the fluid containing SG. Figure 3d shows *M*_w_/*M*_0_ for 0.0035 g/cm^3^ SG at 1.5 M NaCl ramped from pH = 10 to 8.1. The pH threshold at 1.5 M NaCl is pH_t_ = 8.6. The inset shows the threshold [NaCl]_t_ vs pH, with an exponential fit. The latter is an approximate feature of the electrostatic model below.

### Gdn Dialysis and Iso-[Gdn]

Figure 4a,b show the results of LG dialysis against 6 M Gdn, forward and reverse, for scattered intensity (Figure 4a) and *R*_H,ap_ and PI (Figure 4b). Figure 4a shows a large spike in scattering intensity, which subsides and then produces a secondary maximum. The inset to Figure 4a is a computation from the electrostatic model below, and shows how the inter-GLPA energy *U*_*t*_ can have more than one turning points, yielding the nonmonotonic behavior in Figure 4a,b.

**Figure 4:**
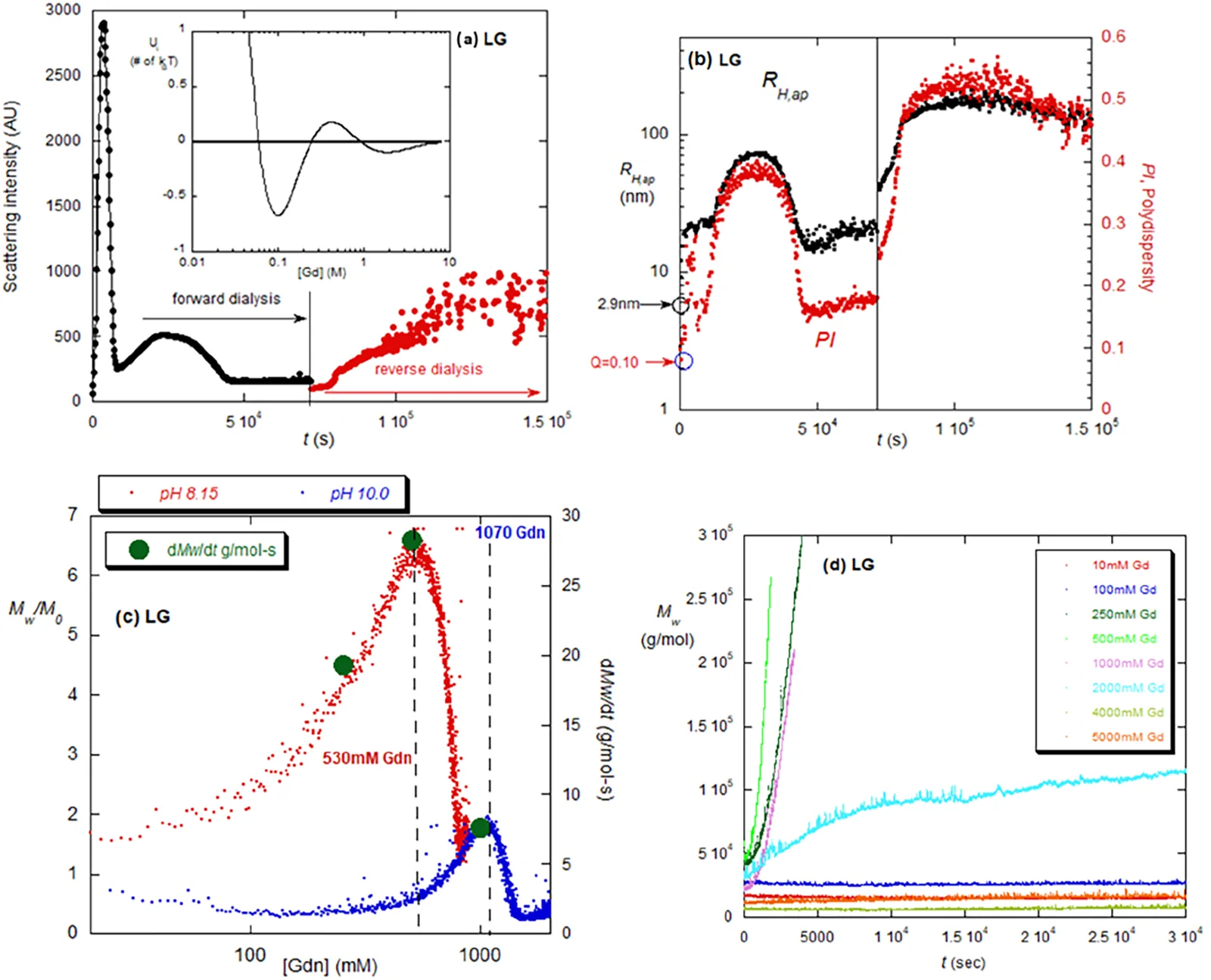
(a) Scattering intensity (AU) for LG 6 M Gdn, forward and reverse dialysis. Inset shows the total energy vs [Gdn], from electrostatic model, below. *z* ^*+*^ = 8, *z*_–_ = 20, *d* = 2 nm, *K* = 15. Its turning points make the double peak plausible. LG was at pH 8.15 and 0.010 g/cm^3^. (b) *R*_H,ap_ and PI for LG 6 M Gdn, forward and reverse dialysis. (c) *M*_w_/*M*_0_ for LG dialysis against Gdn at pH = 8.15 and 10.0, concentration at 0.010 g/cm^3^. The green circles show the molar mass aggregation rates (right-hand axis) from (d); d*M*_*w*_/d*t*. (d) LG *M*_w_ vs *t*, various fixed [Gdn] at pH = 8.15. Aggregation rates for [Gdn] in range of the dialysis are shown as the large circles in (c). Gdn was at pH = 8.15 and 10.0, concentration at 0.010 g/cm^3^.

Figure 4b shows *R*_H,ap_ and PI from DLS. Because of the two-minute DLS cycle time, the early resolution in Figure 4b is not fine enough to see the equivalent spike of Figure 4a, but the immediate jump in *D*_H,ap_ is seen with respect to the circled starting value of *R*_H,ap_ = 5.7 nm. PI = 0.1 at the outset, which is low polydispersity. PI rises in tandem with *R*_H,ap_ to 0.40 on forward dialysis, which is very high, and then falls to 0.16, which is fairly low.

Figure 4c shows Gdn dialysis results for LG in the form of *M*_w_/*M*_0_ vs [Gdn] at pH 8.15 and 10.0. It was run less time than the dialysis in Figure 4a, so the secondary peak is not seen. The upward displacement of the peaks between pH 8.15, peaked at 530 mM Gdn and pH 10.0, peaked at 1070 mM Gdn, is similar to the behavior with NaCl at different pH values in Figure 3c,d. It is again due to increased ionization of acid amino acids and loss of ionization of basic amino acids, making the net charge on the LG more negative, leading to higher repulsion, and the dipole moment lower, which provides weakened attractive potential. The net result is to require more Gdn to screen the higher net charge and to reach the negative dipole potential

Figure 4c also shows the rates, d*M*_w_/d*t*, (right-hand *y*-axis, data are the large circles) obtained from the initial slopes of *M*_*w*_ vs *t* for iso-[Gdn] monitoring at various fixed [Gdn], shown in Figure 4d. The GLPA are stable from 0–250 mM [Gdn], then aggregate up to 2000 mM, at a slower rate. By 4000 mM [Gdn] the GLPA are stable again. It is interesting that the rates, which are a kinetic phenomenon related to the inter-GLPA potential energy barrier, overlap with the dialysis results, which measure instantaneous *M*_w_ and contain no rate information.

Figure 5a shows *M*_w_/*M*_0_ for dialysis of SG at 0.0035 g/cm^3^ against Gdn. The threshold value of Gdn at the abrupt threshold is [Gdn]_t_ = 74 mM ±3.4 mM, averaged over three separate dialyses. Aggregation was so strong in the peak region that the laser intensity for the entire dialysis was reduced from 35 mW to 0.35 mW. SG samples with large decreases in incident laser intensity (controlled by automatically inserted neutral density filters in the Argen) showed turbidity upon visual inspection.

**Figure 5:**
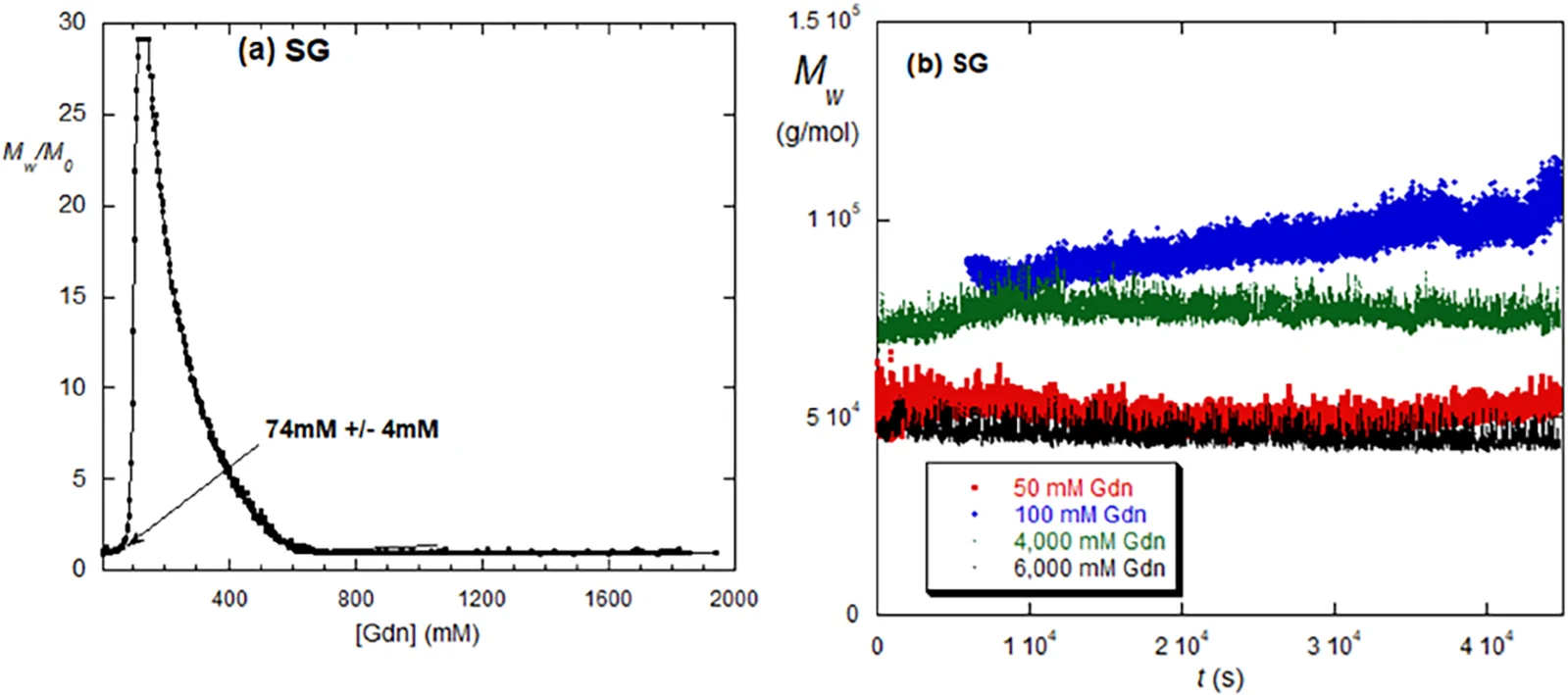
(a) *M*_w_/*M*_0_ for dialysis of SG at 0.0035 g/cm^3^ against Gdn. The laser intensity was reduced from 35 mW to 0.35 mW because the scattering was so intense over the peak region. (b) SG *M*_w_ vs time, various Gdn. The only unstable concentration among these is 100 mM Gdn.

Figure 5b supports the dialysis result. All concentrations are stable, except at 100 mM Gdn, which lies within the unstable peak region of Figure 5a.

There are abrupt threshold concentrations, [NaCl]_t_ and [Gdn]_t_ leading to peak values of *M*_w_, which subside as [NaCl] and [Gdn] increase. Away from the vicinity of the thresholds the GLPA are stable, yet have increased *M*_w_, showing that, away from the thresholds, the dialysis is monitoring equilibrium states reached much faster than the concentration of dialysate is changing. This assertion is examined more closely below.

### Equilibrium vs Nonequilibrium States during Dialysis

As mentioned above, because dialysis is a ramped method–the dialysate gradually entering Fluid 1, containing the GLPA—it is necessary to know whether the light scattering trajectories traced out during dialysis represent equilibrium states at each point. This concern also applies to the well-plate paradigm (e.g., 96 well plates), where it is often assumed that the contents of each well are in equilibrium when measured.

Figure 6a shows the superposition of iso-Gdn data on the dialysis curve for SG vs [Gdn]. The iso-Gdn data in black round circles were constant during 24 h runs; i.e., the dialysis measurements correspond to equilibrium states. However, as the threshold [Gdn]_t_ is approached the iso-Gdn data were not constant in time, and are shown as red circles with upward pointing arrows. The arrows indicate that these are nonequilibrium points, and that *M*_w_ continues to increase. A tentative rule of thumb from this is that light scattering trajectories during dialysis measure true equilibrium states, except that, at any sharp threshold, or within window of high aggregation, the measurements do not represent equilibrium states.

**Figure 6:**
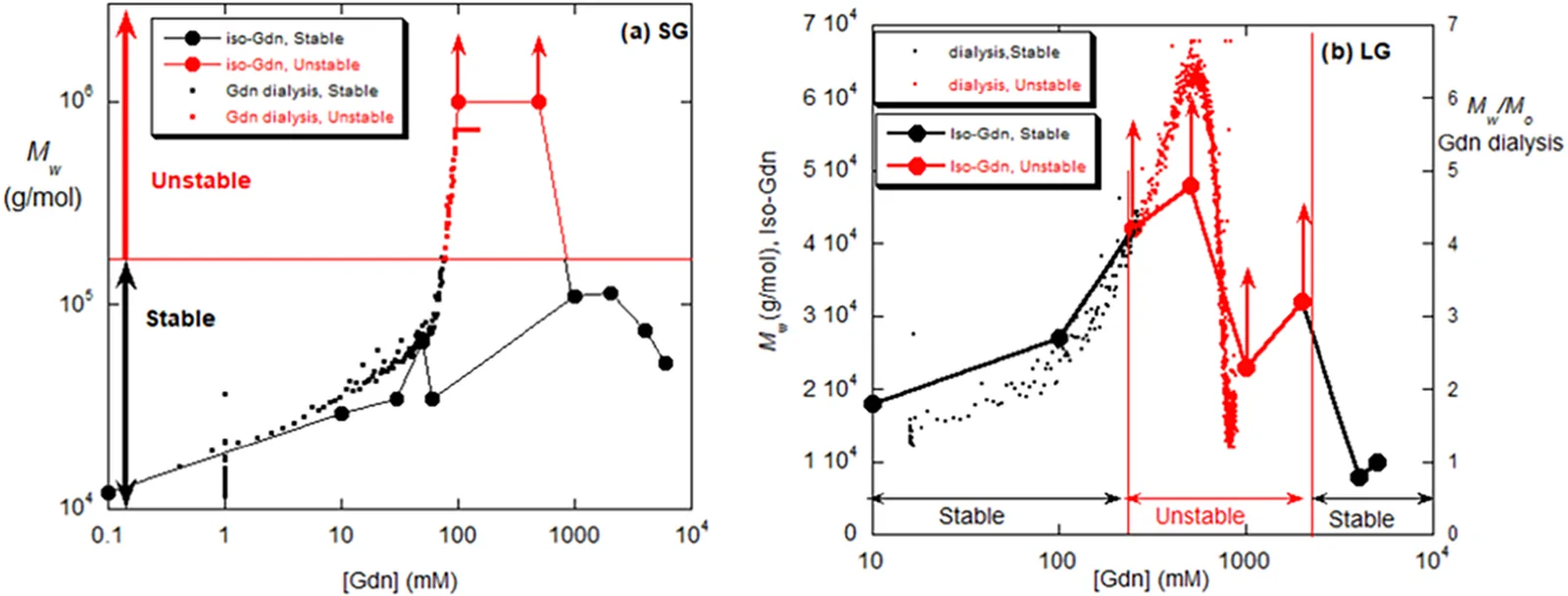
(a) Equilibrium (stable) and nonequilibrium (unstable) portions of the dialysis of SG vs Gdn. Black circles are from Iso-Gdn measurements vs time that were stable and constant, while the red ones are from unstable Iso-Gdn measurements vs time, and use red arrows to indicate the aggregates grow in time. The small black circles are the stable portion of dialysis, while the red ones are in the unstable portion. SG was at 0.010 g/cm^3^. (b) A stability regime representation for LG dialyzed against Gdn. The scheme follows (a), except that the dialysis result is presented as *M*_w_/*M*_0_. LG was at 0.010 g/cm^3^.

Figure 6b shows a similar stability regime representation for LG dialyzed against Gd.

### Temperature Ramps Up and Down (25 °C–65 °C – 25 °C)

Figure 7a–c show temperature up-ramps from 25 °C to 65 °C, and the corresponding down ramps from 65 to 25 °C. SG shows complete reversibility during a complete temperature ramp cycle, and LG shows near-complete reversibility

**Figure 7:**
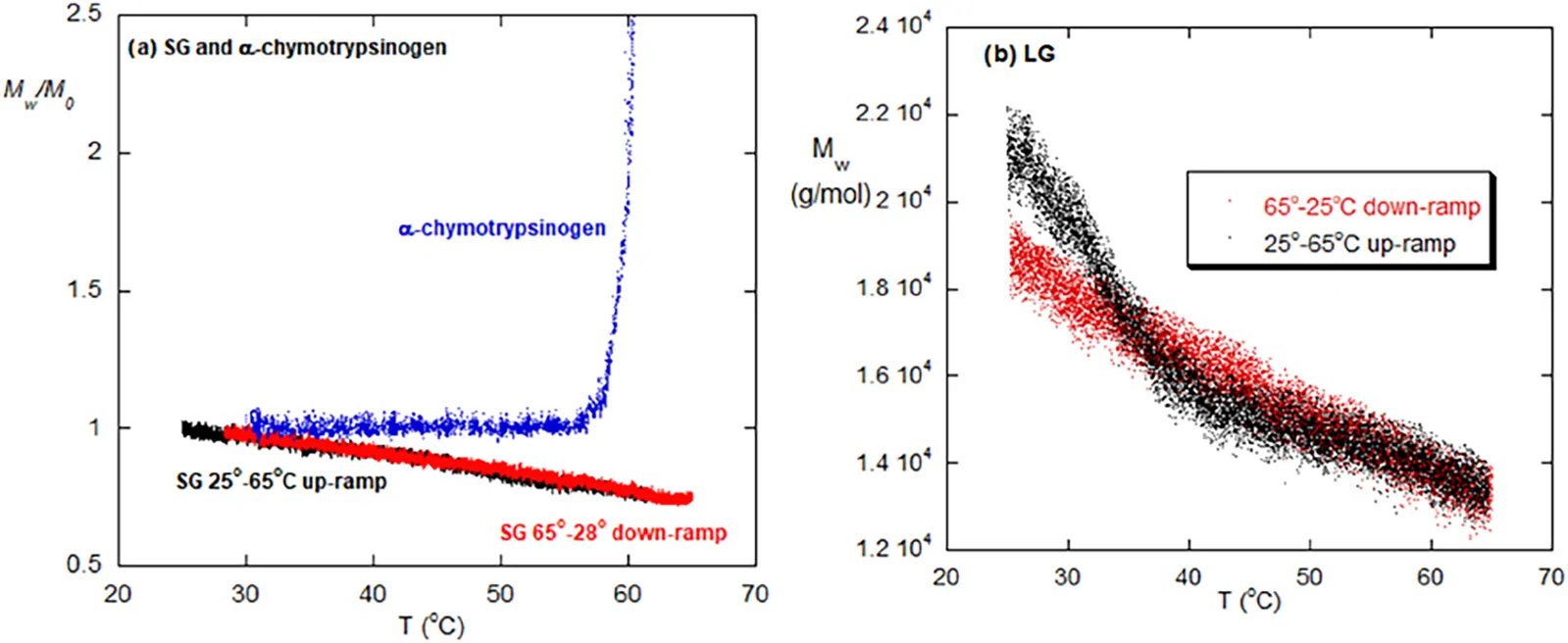
(a, b) Temperature up-ramps, 25 °C–65 °C, and down-ramps, 65 °C–25 °C, for (a) SG, (b) LG. (a) shows massive thermally induced colloidal aggregation of a globular protein, α-chymotrypsinogen. The absence of thermally induced aggregation for GLPA suggest that the classical hydrophobic effect is unlikely to dominate NaCl- and Gdn-induced GLPA aggregation. All samples were at 0.0035 g/cm^3^.

Neither the value of *∂n*/*∂c* for proteins nor the index of refraction of water changes significantly over 25 °C–65 °C, so the decreases in *M*_w_ for SG and LG are real. *M*_w_ for surfactant micelles, such as anionic SDS (sodium dodecyl sulfate) also usually decrease with T.^33^

The contrast in thermal behavior between a globular protein, α-chymotrypsinogen (ACT), and the GLPA is shown in Figure 7a. The ACT goes through colloidal aggregation, where the massive, irreversible aggregation quickly drives it off scale. This illustrates how thermal stress unfolds the ACT, after which the exposed hydrophobic amino acids lead to the aggregation by the classical hydrophobic effect, in which bringing the hydrophobic groups together in an aggregate releases water, thereby increasing its entropy.

None of the GLPA in Figure 7a–c, on the other hand, go through thermally induced aggregation. Rather there is a small, reversible loss of GLPA monomer as T increases for SG and LG. This strengthens the argument that the hydrophobic effect, prevalent in globular protein aggregation, is not the driving force behind GLPA aggregation, and that it is the attractive effect of monopole-dipole and dipole–dipole interactions that cause the aggregation. To summarize, the hydrophobic effect drives the micellization of the GLPA, but orientation-dependent monopole-dipole and dipole–dipole interactions control aggregation of the micelles.

### Kinetic Evolution at Fixed T; Initial Loss of Micelle Mass, Subsequent Gain

As already mentioned, in any ramped method there can be a convolution of the ramp rate and a process rate, such as aggregation rate (AR) of the GLPA. This occurs for the three GLPA here, as all show time-evolution.

Figure 8a,b show *M*_w_ vs *t* for SG and LG, respectively, for a series of fixed temperatures. All show a rapid initial decrease in *M*_w_, albeit with widely different time scales. The time axis is logarithmic, so that the rapid initial decrease is visible. The drop appears to be a lowering of micelle aggregation number, a feature that SG and LG share. After the initial drop, SG and LG reassociate approximately to their original value.

**Figure 8:**
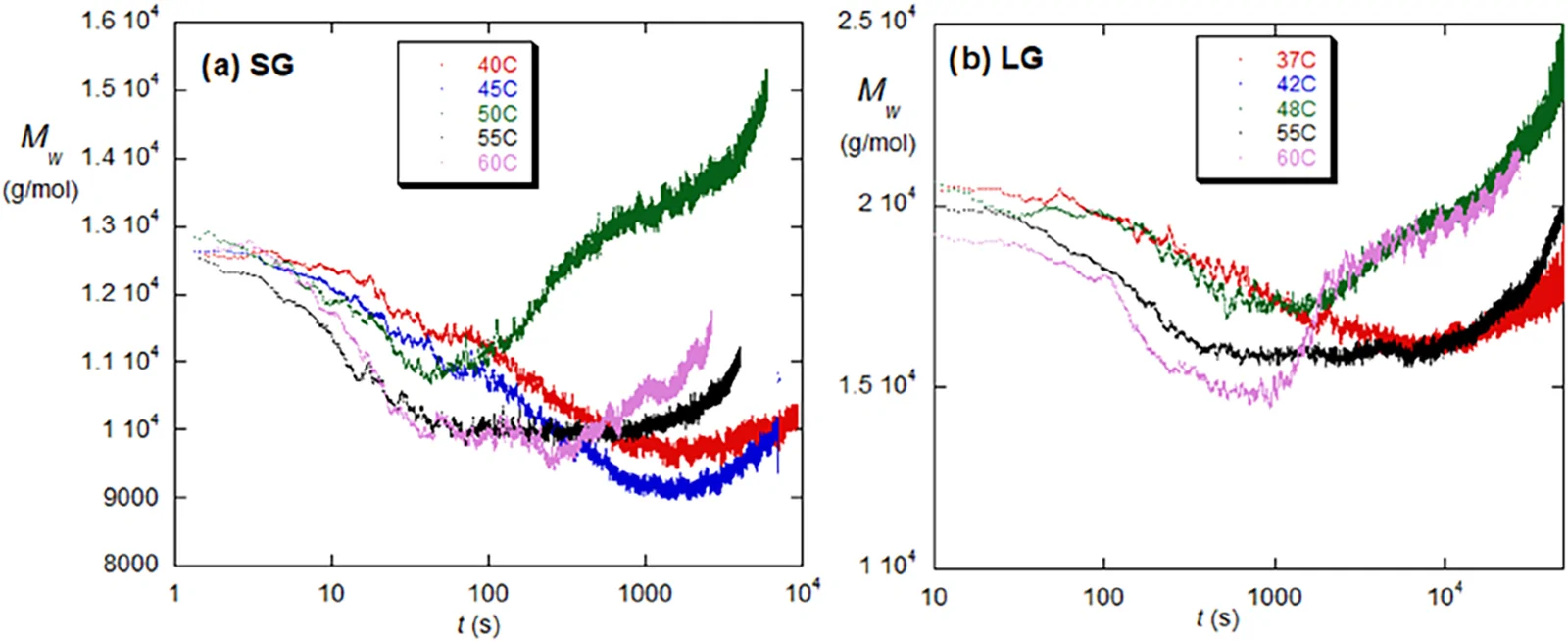
(a, b) *M*_w_ vs *t* for (a) SG, and (b) at a series of fixed temperatures. LG. All samples were at 0.0035 g/cm^3^.

When the minimum values of *M*_w_/*M*_0_ are averaged the average value is [*M*_w_/*M*_0_]_min_ = 0.785 ± 9%. This is consistent for SG and LG. This corresponds to roughly 1 GLPA monomer loss per micelle during this initial period, which can be viewed as a partial dissociation. In the subsequent period the samples essentially regain their starting value *M*_w_/*M*_0_ = 1. This can be considered reassociation.

### Arrhenius Behavior for Dissociation and Reassociation

Figure 9a shows an Arrhenius plot of the absolute values of the dissociation rates, ABS­(DR), where all *DR* are negative. DR is the initial slope of *M*_w_/*M*_0_ vs time. The slopes yields Δ*E*_activation_/*R*, (where *R* is the gas constant) which give similar values; Δ*E*_activation_ = 17.2 kcal/mol ± 14%. This value is in line with reports of computed dissociation energy from surfactant micelles,^34^ and is consistent with the classical hydrophobic theory of micelles.^35,36^

**Figure 9:**
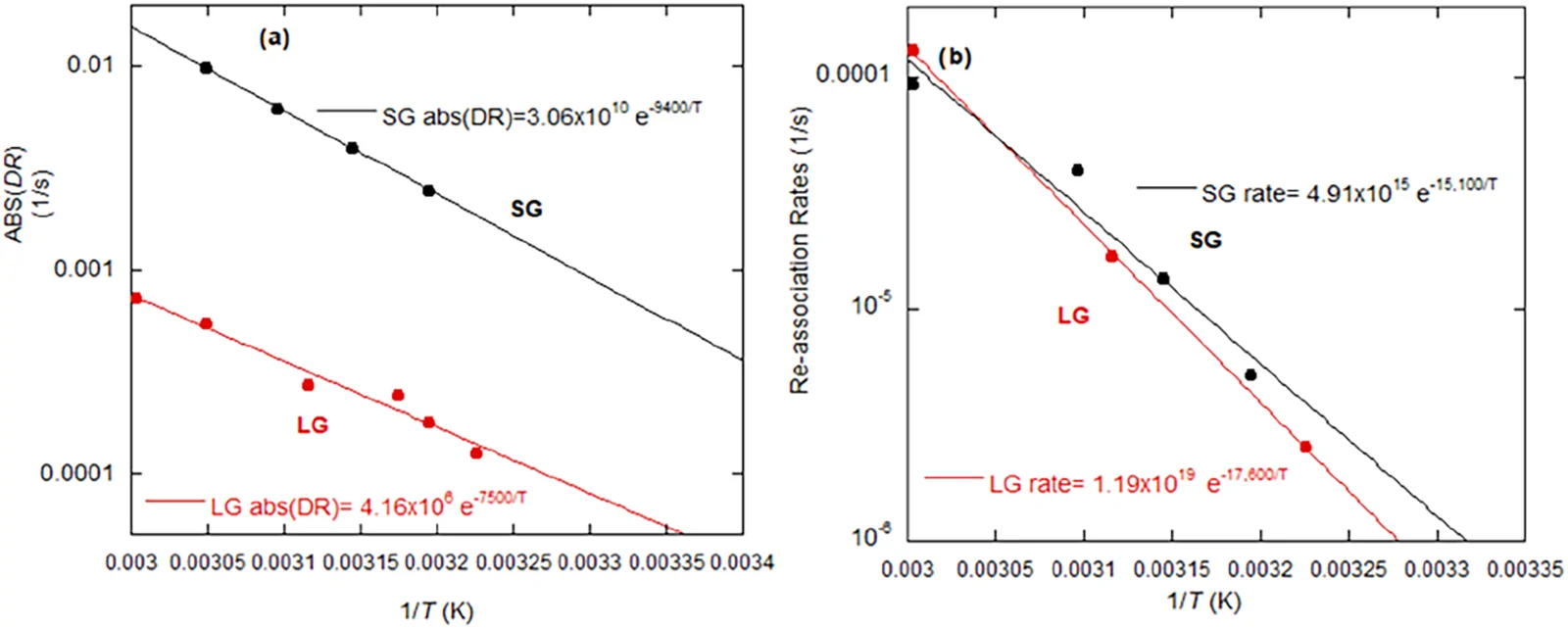
(a) Arrhenius plot for dissociation rates of SG and LG. (b) Arrhenius plot for reassociation rates of SG and LG.

Figure 9b shows an Arrhenius plot of the reassociation rates. SG and LG follow Arrhenius behavior, with rates an order of magnitude slower than the dissociation rates. The average activation energy for SG and LG is 32.0 kcal/mol, about twice the dissociation activation energy.

## Discussion: Energetic Model for Aggregation, Anisotropic Electrostatics

Modeling the aggregation behavior of the GLPA is complex, if all factors and subtleties are considered. The data above can provide grist for computationalists and theorists. Here, the object is to find a relatively simple, yet plausible model for the interactions between these peptides. The model is developed to explain the GLPA stability in terms of dependence on ionic strength and denaturant concentration. It predicts that the ionic-strength dependent instability thresholds should scale as (*Q*_net_/*p*_net_)^2^, and that lowering *Q*_net_ via Gdn^+^ adsorption onto negatively charged amino acids lowers the stability threshold. Both of these effects are experimentally supported. *Q*_net_ is the GLPA micelle net charge and *p*_net_ the scalar value of its dipole moment.

The net charges and dipole moments used in the model are parameters falling within physically plausible ranges. Direct experimental values for the GLPA micelles are not currently available.

### The Electrostatic Model

The existence of a GLPA dipole moment and net charge provides a plausible electrostatic mechanism capable of explaining the observed aggregation trends. The hydrophobic effect is almost certainly a major contributor to GLPA micellization through association of the fatty-acid chains. Hydrogen bonding and π–π interactions between aromatic residues may also contribute to GLPA association and aggregation. The present work does not exclude contributions from these interactions. However, the pronounced dependence of aggregation on ionic strength, pH, and guanidinium concentration suggests that electrostatic interactions play a major role in determining the colloidal stability of the GLPA micelles once formed.. Several publications have assessed the effects of dipole moments on the aggregation of proteins and colloid. The effects of pH on net charge, dipole and quadrupole moments, and the orientations of these have been considered in light of protein data.^37^

The data indicate that electrostatic interactions play a major role in determining GLPA stability, and this allows fundamental electrostatic equations to be used in the model, which can be tested with plausible numbers for net charge and dipole moments, although experimental data for these are scarce. When stability is dominated by the classic hydrophobic effect or π–π stacking, such as in protein and RNA aggregation,^38^ respectively, more phenomenological approaches to describing potentials are needed.

The approach here is as follows: The GLPA have net charge, away from their isoelectric point; IP = 4.9 for LG and IP ∼5.4 for SG. They are polyampholytes with the positive and negative charge groups each clustering together in “patches” on the GLPA surface (Lys, Arg, His are the basic amino acids and Asp and Glu are the acidic amino acids). Clustering of charges in patches for proteins has been studied.^39–41^ As such, the GLPA have net charge *Q*_net_, a net dipole moment *p⃗*_net_, and quadrupole moment, and higher order poles. This means there will be repulsive monopole-monopole (m-m) interactions and, under certain alignments, attractive monopole-dipole (m-d), dipole–dipole (d-d), and lesser terms involving monopole-quadrupole (m-q), and other weak, short-range multipolar interactions. Additionally, there can be attractive H-bonds, hydrophobic effects, and π–π stacking between aromatic side groups (Trp, Phe, Tyr). Scheme 2 is a representation of a GLPA monomer, micelle, net charge, and dipole moment.

**Scheme 2:**
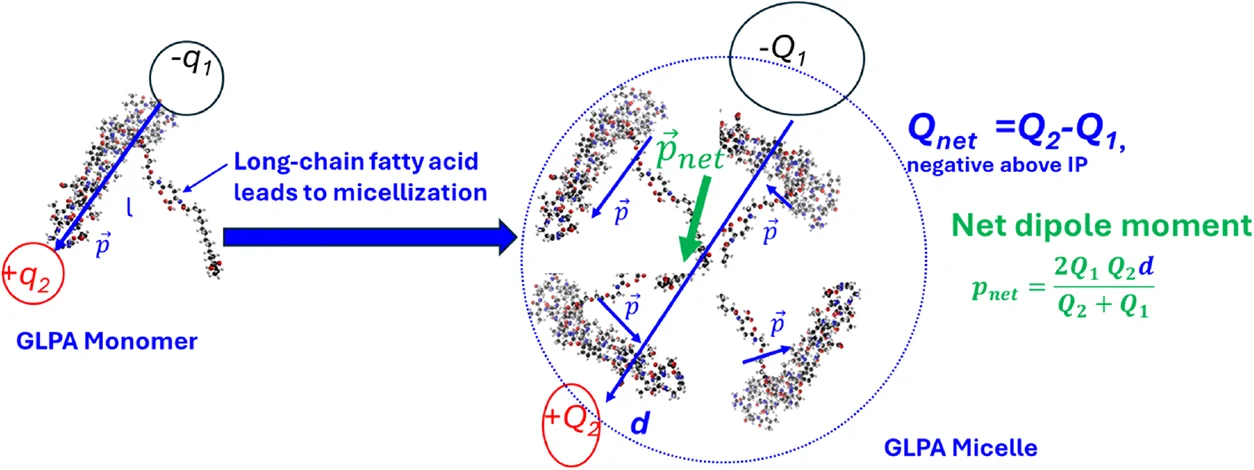
Representation of a GLPA Monomer and Its Corresponding Micelle-like Association^a^

a -*q*_1_ and *q*_2_ are the charges on the monomer (left), separated by distance *l*, and *p⃗* is the monomer dipole moment. On the micelle (right) the effective charges *-Q*_1_ and *Q*_2_ are not necessarily the monomer values multiplied by the number of monomers in the micelle. The distance between the effective charges *-Q*_1_ and *Q*_*2*_ is *d*. The micelle has a net charge *Q*_net_ and dipole moment *p⃗*_net_. In the micelle the vector sum of dipole moments does not cancel out. Although the micelle is conceptually bounded by a sphere in the diagram, there is no assumption of spherical or other morphologies in the model, as it considers morphology-free point charges and point dipoles (See Scheme 3).

This treatment is “morphology-agnostic”, in that whether the GLPA entity is in monomeric, micellar, or aggregate form, it is sufficient for there to be a net charge *Q*_net_, and a net dipole moment *p⃗*_net_ for the model to be applicable, since *Q*_net_ and *p⃗*_net_ are treated as pointlike. Hence, in the following “GLPA” can refer to any of these entities. However, the net charge distribution, dipole moment and distances involved will, nonetheless, define these parameters, and hence the quantitative particulars of each morphological case.

The model considers the interaction between *Q*_net_ and *p⃗*_net_ on one GLPA entity with *Q*_net_ and *p⃗*_net_ on another, at a distance *r* between centers. At 0.0035 g/mL the 27,200 g/mol SG tetrameric micelles (see Table 1) are on average a distance ⟨*d*⟩∼24nm apart, and the LG roughly the same. This estimate is simply based on the number density of particles, *n* (particles/cm^3^)­

$$\left\langle d\right\rangle =1/n^{1/3}$$

where

$$n=N_{A}\left(1/\mathrm{mol}\right)c\left(g/{\mathrm{cm}}^{3}\right)/M_{w}\left(g/\mathrm{mol}\right)$$

Qualitatively, *Q*_net_ between two GLPAs gives a net repulsion which stabilizes the GLPAs against aggregation. The dipole on one interacts with both *Q*_net_ and *p⃗*_net_ on the other. As ionic strength [I], increases the electrostatic shielding allows the two GLPAs to move closer together. At some point, they move close enough that charges on one GLPA are attracted to the opposite sign charges on the other GLPA. This lowers the repulsive m-m barrier, and is formalized as the attractive m-d and d-d terms that come into play. When the barrier is reduced to a few units of *k*_B_ *T*, aggregation can occur.

The actual processes involved in a diffusion-controlled encounter of two GLPAs is quite complex: At long distances, electrostatic potentials are essentially screened out by any added electrolyte, and the individual GLPA translationally and rotationally diffuse without influence from each other, at these concentrations. As the approach gets closer there begins monopole-monopole repulsion, while the electric field of one starts to apply torque to the dipole of the other, a monopole-dipole effect, and vice versa, allowing attraction to also come into play. At close approach the GLPAs face a Coulomb barrier which prevents their aggregation. The dipoles also begin to orient head-to-tail with each other, creating attraction. The alignment is an asymptotic (Langevin) process; i.e., thermal energy keeps them from perfect alignment. If the Coulomb barrier is only a few *k*_B_ *T* in height the GLPAs can thermally surmount the barrier and noncovalently “bind” to each other, that is, aggregate, if there is a net negative potential energy well.

### Considerations on Monomer and Micelle Charge and Dipole Moments

Scheme 2 is an electrostatic representation of the monomer charges -*q*_1_ and *q*_2_ on a GLPA monomer (left). The charges are separated by distance *l* and the monomer has dipole moment *p⃗*. The dipole arises from asymmetric clustering of charged residues along the peptide backbone and incomplete cancellation of monomer dipoles within GLPA micelles. -*Q*_1_ and *Q*_2_ are the effective charges on the micelle (right), separated by distance *d*, and its net charge is *Q*_net_. Also shown is the net dipole moment *p⃗*_net_ on the GLPA, which is the vector sum of each individual dipole, but much less than the scalar sum of the monomer dipole moments, as discussed below.

There have been detailed morphological studies of the micelles with electron microscopy, small-angle X-ray scattering (SAXS), circular dichroism, size exclusion chromatography (SEC), and other methods.^42–44^ However, in the current model, morphology is not a central concern; the only issues of conceptual importance are that the GLPA have positive and negative charges, with a net charge *Q*_net_, and a net dipole moment *p⃗*_net_. The analysis holds for GLPA monomer, micelles, and larger structures.

Importantly, in a GLPA micelle the dipole moments do not cancel out, as could happen in a tightly constrained, idealized situation of a collection of dipoles bound together without any steric or geometrical constraints, and no thermal fluctuations. In spherical geometry with no net charge, the dipoles would be arranged radially (tilt angle = 0) and cancel each other out. With a large enough net charge on each monomer, the dipoles in the micelle will be tangential to the surface (tilt angle 90°), and also cancel. The dipoles would have tilt angles intermediate between 0 and 90° for lesser net charge on each monomer. Because of steric and geometrical constraints, as well as thermal fluctuations, however, the dipoles will not cancel out completely and there will be a net dipole moment *p⃗*_net_, as shown in the micelle in Scheme 2. *p*_net_ will be less than the scalar sum of all the individual monomer dipoles in the micelle, but can still be quite large; e.g., on the order of a single monomer dipole in a small micelle (e.g., octameric). For example, a single monomer with |*q*_1_| = |*q*_2_| = 2_e_ and *d* = 2 nm corresponds to *p*∼200 D (1 D = 3.34 × 10^–30^C-m).

The consistency between GLPA aggregation behavior and the model positing the predominance of monopole-dipole and dipole–dipole attraction offers *prima facie evidence* that the GLPA aggregation behavior is consistent with the presence of a dipole moment, and that monomeric dipoles in the micelle do not cancel out. This does not mean that there is necessarily a fixed, permanent dipole moment associated with the GLPA micelles, as they could be fluctuating, transient, induced, or due to some other spatial charge asymmetry.

### Electrostatic Potential and Interaction Energy in an Electrostatically Screened Environment

The total interaction energy *U_t_* (*r*) between two GLPA, GLPA-A and GLPA-B is the sum of monopole-monopole (m-m), monopole-dipole (m-d), and dipole–dipole (d-d) interactions, as well as higher terms (e.g., monopole-quadrupole), that is

6 $$U_{t}\left(r\right)=U_{m-m}\left(r\right)+U_{m-d}\left(r\right)+U_{d-d}\left(r\right)+U_{\mathrm{higher}\;\mathrm{order}\;\mathrm{terms}}\left(r\right)$$

If *U*_*t*_(*r*) is negative over some regime of *r*, then two GLPAs can bind together to form a bound state (i.e., an “aggregate”), if they reach that regime. If *U*_*t*_(*r*) is positive over all *r*, then two GLPAs cannot form a bound state. Hence, *U*_*t*_(*r*_0_) = 0 is the condition for forming a bound state if *r*_0_ is reached and leads to a regime where *U*_*t*_(*r*) is negative. The height of the Coulomb barrier is the activation energy *U* ^‡^, which controls the aggregation rate, usually in an Arrhenius fashion. In principle, *U*_*t*_(*r*) and *U* ^‡^ are conceptually different; *U*_*t*_(*r*) controls whether a bound state exists and how tightly bound it is, whereas *U* ^‡^ controls the rate of aggregation. However, the two terms are actually related because both terms are affected by the interplay of the positive *U*_m‑m_ and negative *U*_m‑d_ and *U*_d‑d_ terms. Once *U* ^‡^ is reduced to a few units of *k*_B_ *T*, then the GLPAs can thermally surmount the barrier. The overlap of the dialysis data and aggregation rate data in Figure 4c shows how the barrier height (*U* ^‡^) and the well (*U*_*t*_(*r*)), both electrostatic in origin, can be affected in unison by added electrolyte.

The simplest approximation for considering these monopole and dipole interactions is to reduce the GLPA in Scheme 2 to a net point charge *Q*_net_, and net point dipole *p⃗*_net_ at the origin at a distance *r* from the second GLPA, which is portrayed in Scheme 3, along with the relevant angles. The electrostatic potential at distance *r* from a single charge of *Q*_net_ for this arrangement is given in eq 7. The total electrostatic potential *V*_*t*_(*r*) for this charge can be represented with the multipole expansion, including the electrostatic shielding given by the Debye parameter κ,^45,46^ given in eq 8. In MKSA units the multipole expansion is

7 $$V_{t}\left(r\right)=\frac{Q_{\mathrm{net}}e^{-\kappa r}}{4\pi \varepsilon r}-\frac{e^{-\kappa r}}{4\pi \varepsilon }\left(1+\kappa r\right)\frac{{\mathrm{p⃗}}_{\mathrm{net}}\cdot \mathrm{r̂}}{r^{2}}+\frac{e^{-\kappa r}}{4\pi \varepsilon }\frac{\mathrm{r̂}\cdot \mathrm{T̿}\cdot \mathrm{r̂}}{r^{3}}+...O\left(\frac{1}{r^{4}}\right)$$

where the first term on the right-hand side is the monopole potential, the second term is the dipole potential, the third is the quadrupole term, where *T̿* is the quadrupole tensor, and the last term is for octupole and higher terms. *r̂* is the radial unit vector, pointing outward from the point charge. In general, there are nonzero quadrupole and higher moments. Here, the expansion will be cut off above the dipole term, the quadrupole term being usually, but not always negligible compared to the monopole and dipole terms. Some studies have delved into protein quadrupole and higher moments.^37,47^

**Scheme 3:**
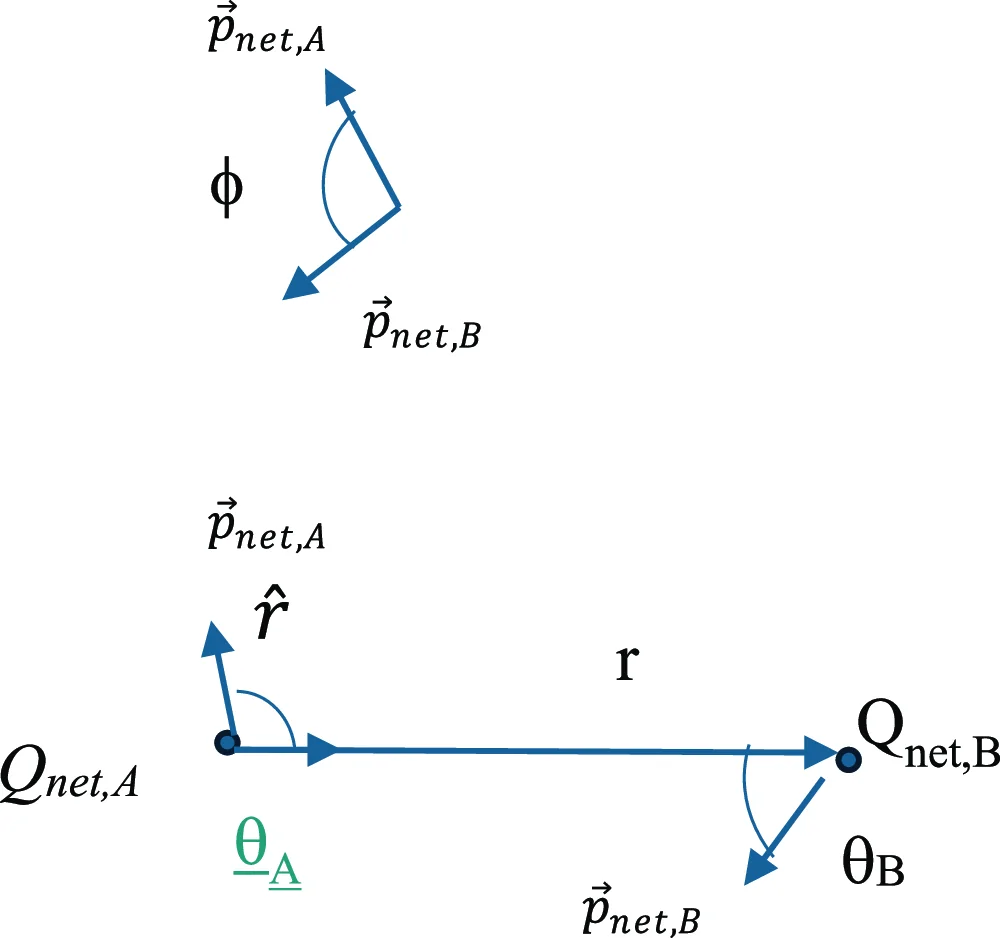
Reduction of the GLPA Micelle in Scheme 2 to a Point Charge and Point Dipole^a^

a The scheme shows the geometry for two GLPAs, A and B, showing the net charges, the dipoles, the separation *r*, the unit direction vector *r̂*, the angles θ_A_ and θ_B_, and, separately, ϕ the angle between the two dipoles.

The Debye screening parameter is given, for a monovalent electrolyte, (in MKSA units) by

8 $$\kappa ={\left(\frac{2\rho_{0}e}{\varepsilon k_{B}T}\right)}^{1/2}$$

where ρ_0_ is the bulk charge density (C/m^3^) of added electrolyte, Gdn or NaCl (both monovalent) in this work (ρ_0_ is the charge density of positive charge, which is equal and opposite in sign to the negative charge density that reflects electroneutrality in the simple bulk electrolyte solution), *e* is the elementary charge, *z* is the valence for symmetric electrolytes (*z* = 1 for Gdn and NaCl), ε = ε_0_ *Di*, where ε_0_ = 8.85 × 10^–12^ C^2^/N-m^2^ is the permittivity of free space and *Di* is the dielectric constant of the solution (∼80 for H_2_O at STP), k_B_ is Boltzmann’s constant (1.38 × 10^–23^ J/K), and *T* is the temperature in Kelvin. Note that κ is very weakly dependent on *T* near 25 °C; it decreases only 8% from 25–75 °C. Expressing κ in the more general form using ionic strength [I] (mol/L), for any valence and symmetry/asymmetry of the electrolyte

9a $$\kappa^{2}=\frac{2000N_{A}e^{2}}{\varepsilon k_{B}T}\left[I\right]$$

where

9b $$\left[I\right]\left(M\right)=\frac{1}{2}\sum_{i}{\left[X\right]}_{i}z_{i}^{2}$$

where [X]_*i*_ is the molar concentration of ion species i, and *z*_*i*_ is its valence. [Gdn] and [NaCl], being monovalent, are used for [I] in their respective cases.

It is convenient to give a rapidly calculable estimate of the Debye screening length κ^–1^ at 25 °C.

9c $$\kappa^{-1}\left(\mathrm{nm}\right)\approx \frac{0.304}{\sqrt{I\left(M\right)}}$$

The total electrostatic energy U_t_(r), at any given set of orientations of the dipoles with respect to the unit vector *r̂* in Scheme 3, and to each other, ϕ, is given by

10 $$U_{t}\left(r\right)=\frac{Q_{\mathrm{net},A}Q_{\mathrm{net},B}}{4\pi \varepsilon }\frac{e^{-\kappa r}}{r}-\frac{(1+\kappa r)e^{-\kappa r}}{4\pi \varepsilon r^{2}}\left[Q_{\mathrm{net},A}\left({\mathrm{p⃗}}_{\mathrm{net},B}\cdot \mathrm{r̂}\right)+Q_{\mathrm{net},B}\left({\mathrm{p⃗}}_{\mathrm{net},A}\cdot \mathrm{r̂}\right)\right]+\frac{e^{-\kappa r}}{4\pi \varepsilon r^{3}}\left[\left(1+\kappa r\right){\mathrm{p⃗}}_{\mathrm{net},A}\cdot {\mathrm{p⃗}}_{\mathrm{net},B}-\left(3+3\kappa r+{\left(\kappa r\right)}^{2}\right)\left({\mathrm{p⃗}}_{\mathrm{net},A}\cdot \mathrm{r̂}\right)\left({\mathrm{p⃗}}_{\mathrm{net},B}\cdot \mathrm{r̂}\right)\right]$$

where the first term on the left is *U*_m–m_(*r*), the second is *U*_m–d_(*r*), and the third is *U*_d–d_(*r*). *r̂* is the unit vector shown in Scheme 3. *p⃗*_net,A_·*p⃗*_net,B_ = *p*_net,A_ *p*_net,B_ cos ϕ. The monopole-quadrupole energy *U*_m‑q_ is not included, but it may reach near the order of magnitude of *U*_d‑d_ under certain conditions such as close approach and near the isoelectric point.

Equation 12 is highly dependent on the dipoles’ orientations, θ_A_ and θ_B_, with respect to *r̂*, and to each other, ϕ. Above the GLPA isoelectric point, both *Q*_net,A_ and *Q*_net,B_ are negative so let *Q*_net,A_ *= Q*_net,B_ *= Q*_net_. Then, (*p⃗*_net,B_·*r̂* + *p⃗*_net,A_·*r̂*) must be negative in order for the second term, which diminishes as 1/*r* ^2^, to be attractive. The third term, *U*_d‑d_, which is much shorter range and diminishes as 1/*r* ^3^, will be negative as long as

11a $$\frac{\left(1+\kappa r\right){\mathrm{p⃗}}_{\mathrm{net},A}\cdot {\mathrm{p⃗}}_{\mathrm{net},B}}{(3+3\kappa r+{\left(\kappa r\right)}^{2})\left({\mathrm{p⃗}}_{\mathrm{net},A}\cdot \mathrm{r̂}\right)\left({\mathrm{p⃗}}_{\mathrm{net},B}\cdot \mathrm{r̂}\right)}<0$$

In order for a bound state to exist (*U_t_* ≤ 0), the first term must be less than the sum of the second and third terms, and the range of *r* for which this holds defines the distance limits of the negative potential energy well.

As mentioned above, there are several factors affecting orientation as the micelles approach each other, so that eq 12 at any instant involves different average orientations in the scalar (dot) product terms as a function of *r*; i.e., ⟨*p⃗*_net_·*r̂*⟩ = ⟨*p⃗*_net_·*r̂*⟩(*r*), ⟨*p⃗*_net_·*p̂*_net_⟩ = ⟨*p⃗*_net_·*p⃗*_net_⟩(*r*). It is best left to a more detailed, computational or simulation analysis to understand how the orientations, and eq 12, change as a function of *r*. In light of this complication, no attempt is made here to give a general approximation to the r-dependence of *U*_*t*_(*r*).

When the GLPA are far apart the orientations are thermally randomized. As they approach each other more closely, however, the oppositely charged patches, which constitute *p⃗*_net_ will minimize their energy when the positive and negative patches attract each other maximally, which occurs when *p⃗*_net,A_ and *p⃗*_net,B_ are aligned head-to-tail, and both are antiparallel to *r̂* (θ = 180°), that is

11b $${\mathrm{p⃗}}_{\mathrm{net},A}\cdot \mathrm{r̂}=-p_{\mathrm{net},A},{\mathrm{p⃗}}_{\mathrm{net},B}\cdot \mathrm{r̂}=-p_{\mathrm{net},B}$$

11c $${\mathrm{p⃗}}_{\mathrm{net},A}\cdot {\mathrm{p⃗}}_{\mathrm{net},B}=p_{\mathrm{net},A}p_{\mathrm{net},B}$$

While this condition is only asymptotically approached, as per the Langevin equation, it becomes better the closer the GLPA get to each other. Outside, however, where dipoles are weakly aligned, eq 12 with this condition will overestimate the attraction and hence underestimate the *U_t_* (*r*) and *U* ^‡^. Another point favoring increasing, Langevin-like alignment of dipoles as GLPA approach each other, is that computations show (not reproduced here) that the time for a dipole to orient in a plausible E-field due to a GLPA molecule of 8.3 × 10^4^ V/cm at 4 nm away, dipole moment of 100 D, and net charge 10*e*, orients in a time over an order of magnitude less than the time it takes for rotational diffusion to dis-align approaching dipoles.

Since the GLPA are presumed identical in *Q*_net_ and *p*_net_, *Q*_net,A_ = *Q*_net,B_ = *Q*_net_, *p*_net,A_ = p_(net,B)=p_net. With these simplifications, and the conditions in eqs 14,15 for minimum energy, *U*_*t*_(*r*) in eq 12 becomes

12 $$U_{t}\left(r\right)=\frac{e^{-\kappa r}}{4\pi \varepsilon }\left[\frac{Q_{\mathrm{net}}^{2}}{r}-\frac{2\left|Q_{\mathrm{net}}\right|p_{\mathrm{net}}\left(1+\kappa r\right)}{r^{2}}-\frac{p_{\mathrm{net}}^{2}\left[{\left(\kappa r\right)}^{2}+2\kappa r+2\right]}{r^{3}}\right]$$

The first term in the bracket, $\frac{Q_{\mathrm{net}}^{2}}{r}$ is positive, repulsive and long-range, whereas the second (m-d) and third (d-d) terms are negative and attractive, and drop off much more rapidly than *U*_m‑m_. The use of absolute value |*Q*_net_| guarantees the *U*_m‑d_ term is negative in this orientation.

### Condition at Which a Bound Aggregate State Exists; *U_t_* (*r*_0_)=0

The model now focuses on whether a bound (aggregate) state exists under varying ionic strength and pH, while a kinetic study involving *U* ^‡^ is left to separate work. The condition for *U*_*t*_(*r*_*0*_) = 0 can be found analytically from eq 16.

13 $$Q_{\mathrm{net}}^{2}r_{0}^{2}-2\left|Q_{\mathrm{net}}\right|p_{\mathrm{net}}\left(1+\kappa r_{0}\right)r_{0}-p_{\mathrm{net}}^{2}\left[{\left(\kappa r_{0}\right)}^{2}+2\kappa r_{0}+2\right]=0$$

where *r*_0_ is the interparticle distance at which *U*_*t*_(*r*)=0. This quadratic can be solved for *r*_0_ in terms of |*Q*_net_|, *p*_net_, and κ. However, the interest here is to find at what threshold ionic strength, [I]_t_, *U*_*t*_([I]_t_) = 0, so *r*_*0*_ is not of direct interest. Hence, a scaling level condition is used for aggregation, considering it reached when *r*_0_ is equal to the threshold screening length 1/κ_*t*_. This heuristic takes the characteristic length of the system as the scale for the *U*_*t*_(*r*_*0*_)=0 condition, and is predictive of trends, not exaxt. It also permits an analytical solution, and turns eq 17 into

14 $$\kappa_{t}^{2}+\frac{4}{5}\frac{\left|Q_{\mathrm{net}}\right|}{p_{\mathrm{net}}}\kappa_{t}-\frac{Q_{\mathrm{net}}^{2}}{5p_{\mathrm{net}}^{2}}=0$$

The positive (physical) root to eq 18 is

15a $$\kappa_{t}=\frac{\left|Q_{\mathrm{net}}\right|}{5p_{\mathrm{net}}}$$

This is the close approach value. When the particles are farther apart there is little dipole alignment so the attractive term is less, and can even be repulsive. The maximum repulsive term is when θ = 0°. When this is substituted into eq 12, and the analogous quadratic equation from eq 12 is solved

15b $$\kappa_{t}=\frac{\left|Q_{\mathrm{net}}\right|}{p_{\mathrm{net}}}$$

Using eqs 8 and 9 (for monovalent salt), eqs 19 and 20 can be expressed in terms of the threshold ionic strength, [I]_t_, at *U*_*t*_(*r*_*0*_) = 0, using the micelle’s net number of elementary charges *z*_net_ and its net dipole’s number *n*_net_ of Debyes (D), where 1D = 3.34 × 10^–30^ C-m.

16a $$\begin{array}{ll} {\left[I\right]}_{t}\left(M\right)=3.81\times 10^{-21}{\left(\frac{Q_{\mathrm{net}}}{p_{\mathrm{net}}}\right)}^{2}∼8.7{\left(\frac{z_{\mathrm{net}}}{n_{\mathrm{net}}}\right)}^{2} & \mathrm{inside the well} \end{array}$$

whereas

16b $$\begin{array}{ll} {\left[I\right]}_{t}\left(M\right)∼214{\left(\frac{z_{\mathrm{net}}}{n_{\mathrm{net}}}\right)}^{2} & \mathrm{near the well},\mathrm{outside} \end{array}$$

where

17a $$Q_{\mathrm{net}}=z_{\mathrm{net}}e$$

17b $$p_{\mathrm{net}}=n_{\mathrm{net}}D$$

where *n*_net_ is the number of Debyes in the dipole moment. In both limits the power law is the same, ${\left(\frac{z_{\mathrm{net}}}{n_{\mathrm{net}}}\right)}^{2}$ , whereas the prefactor varies between the limits by a factor of 25.

Plausible values for *Q*_1_, *Q*_2_, and *p*_net_, using eq 22, are discussed below, which can account for threshold concentrations of electrolyte, [I]_t_ ([NaCl]_t_ and [Gdn]_t_). (*Q*_1_ and *Q*_2_ are always represented as positive numbers here. *-Q*_1_ indicates that the negative patch corresponding to *Q*_1_ is negative. *Q*_net_ can be positive of negative, but is always negative above the GLPA isoelectric point). Data in Figures 3a,c,d, 5a, and 6a show sharp aggregation thresholds, whereas Figures 4a and 6b show peaks, rather than sharp thresholds. In the latter case, the value of [I] at the peak maximum is taken to correspond to the *U*_*t*_(*r*_*0*_)=0 conditions, and is designated by [I]_p_.

These considerations are for dimerization of micelles when their thermal energy surpasses the Coulomb barrier. The details of aggregation between initial dimers and monomers of micelles and other dimers may depend on the structure of the aggregates. The dimerization criteria here provide a basis for the initial aggregation, which can become massive colloidal aggregation in some instances; Figures 3a,c,d, 4a, and 5a.

### Summary of the Assumptions in the Electrostatic Model

i. The GLPA are treated as pointlike objects with net charge *Q*_net_ and dipole moment *p⃗*_net_, so that the electrostatic potential is the sum of monopole and dipole terms. Quadrupoles and higher moments are neglected. The interactions between two GLPA are then m-m, m-d, and d-d, where the first is repulsive and the next two are attractive under favorable orientations. This approach is independent of GLPA morphology, although the particulars of each morphological case—charge distribution, dipole moment and approach distances—will quantitatively affect the values of these parameters.
ii. The criterion of *U*_*t*_(*r*_*0*_) = 0, separating bound and unbound states was used for determining the onset of aggregation, heuristically approximated to occur when r_0_ reaches the threshold Debye length; i.e., when *r*_0_=1/*κ*_t_. The threshold ionic strength [I]_t_, or the ionic strength at peak at which this occurs is then found from this κ_t_.
iii. Inclusion of the quadrupole moment term -representing higher-order anisotropic interactions- would, under favorable alignment, further increase binding among the micelles, but these secondary effects are not central to the current analysis, and quadrupole and higher moment effects are treated as negligible.

### Consideration of Hydrophobic and other Attractive Effects

While the electrostatic model, via the *U*_m‑d_ and *U*_d‑d_ terms, captures the principal trends in the aggregation experiments, there are other attractive effects that can enhance the aggregation. These include H-bonds, π–π stacking (quantum mechanical), and the classical hydrophobic effect (entropy increase of displaced water). Interestingly, the latter is the chief mechanism driving aggregation of unfolded globular proteins, and electrostatic and π–π interactions are secondary. The GLPA micelles do not resemble globular proteins and do not unfold and aggregate the way most globular proteins do. Figure 7a explicitly shows no thermally induced aggregation, in contrast to the globular protein α–chymotrypsinogen.

In RNA, π–π stacking is found to be the principal driver of aggregation,^37^ which is due to the high density of π-bonds in their heteroaromatic purine and pyrimidine rings. In contrast, the GLPA only have a few aromatic amino acids; Phe, Tyr, and Trp. The sharp ionic strength thresholds and pH-dependent shifts in aggregation behavior are electrostatic signatures, not those of π–π stacking.

H-bonds can occur between amino acids. However, H-bonds are very short-range, around 0.1–0.3 nm, so do not contribute as much as the overall longer range electrostatic effects.

Taken together, classical hydrophobicity, H-bonds and π–π stacking should help stabilize the aggregates, once formed, but the formation of aggregates from micelles is due primarily to electrostatic interactions.

### Net Charge Reduction due to Binding of Gdn^+^ on Acidic Amino Acids in OP

Figure 10a shows that the aggregation threshold for Gdn, at 0.074M, is far less than the 0.670 M threshold for NaCl. This results because the cationic species of Gdn, Gdn^+^ (used to represent the guanidinium cation), has its charge delocalized over its planar structure. If ionic strength were the only relevant effect on aggregation at these relatively low concentrations of Gdn and NaCl, the dialysis aggregation data should be close to each other. The fact that the Gdn threshold is an order of magnitude lower than that of NaCl strongly suggests a charge neutralization effect is in play. This mechanism is different from the chaotropic ability of Gdn to disrupt H-bonds and hydrophobic associations, which normally requires [Gdn]>1M. The charge neutralizations occur at [Gdn]<1M.

**Figure 10:**
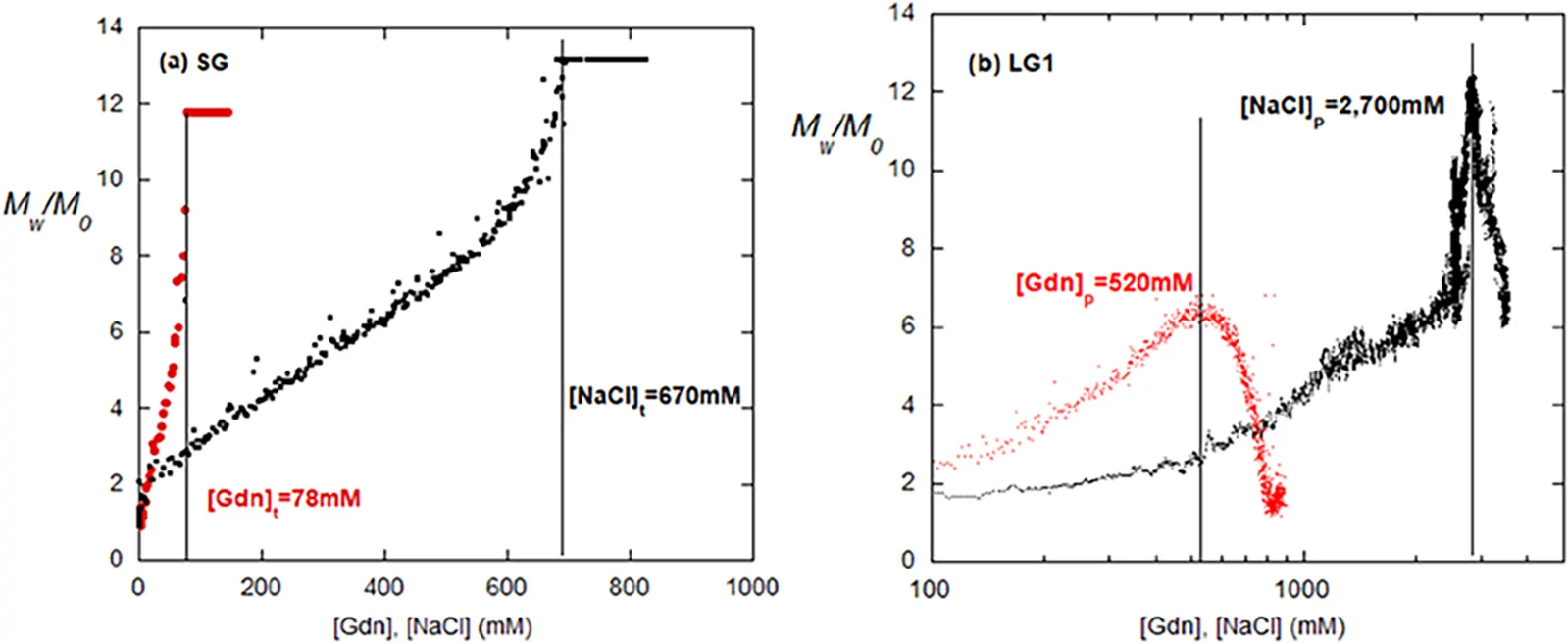
(a) Contrast of thresholds [Gdn]_t_ and [NaCl]_t_ for SG. [Gdn]_t_ ≪ [NaCl]_t_ because of the partial neutralization of negative charge by Gdn adsorption onto deprotonated acidic amino acids. The horizontal bars are the saturation of the scattering photodetector. SG was at 0.010 g/cm^3^. (b) Contrast of peak values [Gdn]_p_ and [NaCl]_p_ for LG at pH = 8.15. The trends for Gdn and NaCl are similar to those in (a), but the values are quite different. LG was at 0.010 g/cm^3^.

There is extensive evidence that, due to its planar nature and delocalized positive charge, Gdn^+^ can bind to groups with COO^–^ moieties.^48,49^ This means that Gdn^+^, even at low concentration, can bind to negatively charged amino acids, effectively canceling their charge. This is a major effect because the stabilizing monopole-monopole repulsive interaction is proportional to the net charge squared; *Q*_net_^2^. Hence, anything that can lower this charge directly will powerfully affect aggregation, making it occur at much lower ionic strength than mere electrostatic screening due to ionic strength. At the same time, however, lowering *Q*_net_ can also lower the attractive *U*_m‑d_ term as well as the *U*_d‑d_ term, since *p*_net_ depends on *Q*_net_, and how it is composed of *Q*_*1*_, which is the negative GLPA patch, and *Q*_*2*_ the positive patch.

Consider

18 $$Q_{\mathrm{net}}=Q_{2}-Q_{1}=e\left(z^{+}-{\mathrm{fz}}^{-}\right)$$

where *z* ^+^ and *z* ^–^ are the number of positive and negative elementary charges on the GLPA micelles, respectively, at a given pH; z *= Q*/*e*. Both *z* ^*+*^ and *z* ^*–*^ are positive. *f* is the fraction of original negative charges left after Gdn^+^ binds to negative amino acids, which depends on [Gdn].

As far as finding *f*, an approximation is to use the Langmuir binding isotherm, which gives the fraction of negative binding sites on the GLPA which are bound to Gdn^+^, which is (1-*f*), and has the form

19a $$\left(1-f\right)=\frac{K_{\mathrm{ap}}\left[\mathrm{Gd}\right]}{1+K_{\mathrm{ap}}\left[\mathrm{Gd}\right]}$$

where, because Gdn-HCl is monovalent and fully dissolved, or

19b $$f=\frac{1}{1+K_{\mathrm{ap}}\left[\mathrm{Gdn}\right]}$$

where *K*_ap_ is the effective, phenomenological equilibrium binding constant. *K*_ap_ depends on pH because more acid amino acids deprotonate as pH increases, giving more binding sites, and increasing *K*_ap_. [I] can influence *K*_ap_ because it can affect amino acid p*K*_a_’s, but the pH effect is larger.

Figure 10a for SG shows the charge neutralization effect leads to the dramatic difference in [I]_t_ between [Gdn]_t_=0.074 M and [NaCl]_t_=0.670M; nearly a factor of 10. The net charge and dipole moment of the GLPA does not change as [NaCl] increases (*f* = 1), there is just net charge and dipole screening. In the charge neutralization caused by Gdn^+^ binding, both the net charge and dipole weaken. This rapidly lowers the repulsive *U*_m‑m_ while the dipole also weakens, the net effect being to require less [Gdn] than [NaCl] at the aggregation threshold.

Figure 10b shows the charge neutralization effect for LG in the buffer at pH 8.15. The peak ionic strengths [I]_p_ for Gdn and NaCl are again well separated, but occur at significantly higher values than for SG in Figure 10a; [Gdn]_p_ = 520 mM and [NaCl]_p_ = 2700 mM.

The behavior in Table 2 shows that, for SG, as pH increases from 6.7 to 10.0 [NaCl]_t_ is a factor of 4× greater, whereas [Gdn]_t_ is unmeasurably high, [Gdn]_t_ > 6M. GLPA has a higher negative charge at pH = 10.0 and correspondingly lower dipole moment, neither of which substantially change with increasing NaCl. Screening increases with NaCl, and more is needed to overcome *U*_m‑m_. In contrast, when dialysis with Gdn is done at pH = 10.0 both the net charge and dipole moment decrease, as increasing Gdn lowers the net charge but also rapidly diminishes the dipole, such that the net attractive terms *U*_m‑d_ and *U*_d‑d_ decrease in strength, and since these latter are the driving force for aggregation, no aggregation occurs, at least up to 6M. This case shows how both Gdn^+^ binding and increasingly ionized acidic amino acids at higher pH can combine to weaken the SG dipole enough to completely avoid aggregation under Gdn.

**Table 2:** Estimates of *f* and *K*_ap_ for SG at pH 6.7, 8.6, 10.0, and LG at pH 8.15 and 10.0^a^,^b^

pH	[Gdn]_t_ (M)	[NaCl]_t_ (M)	$B=\sqrt{\frac{{\left[\mathrm{Gd}\right]}_{t}}{{\left[\mathrm{Na}\mathrm{Cl}\right]}_{t}}}$	*Q*_1,HH/e_	*Q*_2,HH_/e	*Q*_net,HH_/e (*f* = 1)	*f*	*K*_ap_ (M^–1^)	*p*_monomer_ (*D*) (*f* = 1) eq 38	|*z*_net_/*n*_net_|
			Semaglutide (SG)							
6.7	0.078	0.68	0.338	4.97	3.12	–1.85	0.536	4.54	115.3	0.016
8.6	NA	1.50	NA	5.03	2.20	–2.83	NA		64.1	0.044
10	>6.0	3.13	<0.5	5.44	2.00	–3.44	NA		51.5	0.066
	[Gdn]_p_	[NaCl]_p_ (M)	Liraglutide (LG)							
8.15	0.52	2.78	0.432	5.00	1.42	–3.58	0.545	10.7	30.1	0.119
10.0	1.05	>1.6	0.81 (max value)	5.40	1.01	–4.39	0.82 (max)	2.74 (min)	15.2	0.289

a *Q*_1,HH_/e and *Q*_2,HH_/e are the Monomer Values from Application of the Henderson-Hasselbach Equation Based on p*K*_a_’s of Free Amino Acids.

b For the GLPA monomer dipole moment, d ∼ 1.0 nm. p/D is the monomer dipole moment in D.

There is no evidence that simple cations, such as Na+, bind specifically to the protein surface. Rather, they form a nondirectional, nonbound, diffuse cloud of electrostatic shielding.

### Computation of p: Charge-Magnitude Weighted Origin (CMWO) Position for *p⃗*

Because *Q*_net_ ≠ 0, except at the isoelectric point, *p⃗* is dependent on where the origin of the coordinate system is chosen; it is a gauge choice, so there are several options. In order to reduce the dependence on an arbitrary origin, the charge-magnitude-weighted-origin (CMWO) will be used, which will be important for *Q*_net_ ≠ 0; i.e., for all the pH’s of interest. Its virtue is that it avoids overweighting a large charge compared to a much smaller charge if the geometric origin is chosen, while capturing the proper limits for *Q* = 0, and for *Q*_1_ = *Q*_*2*_. (Again, *Q*_1_ and *Q*_2_ are the absolute magnitudes of the charges. *Q*_1_ is consistently treated as the negative charge, and so appears as *-Q*_1_ in equations where the sign is vital).

Scheme 4 shows the representation of an SG micelle as positive charge *Q*_*2*_ and negative charge, *-Q*_1_, whose effective charge is −*fQ*_1_, where *f* is the fractional number of negative amino acids not neutralized due to Gdn^+^ binding. At a given pH *Q*_1_ and *Q*_2_ are constant. The distance between the negative and positive charge patches is *d*, assumed constant, and the CMWO is defined by the condition

20 $$-{\mathrm{fQ}}_{1}d_{1}+Q_{2}d_{2}=0$$

where *d*_1_ and *d*_2_ are the distances of *Q*_1_ and *Q*_2_ from the CMWO, respectively, and *d* = *d*_1_ + *d*_2_. With this

21 $$p=fQ_{1}d_{1}+Q_{2}d_{2}$$

where

22 $$d_{2}=\frac{fQ_{1}d_{1}}{Q_{2}}$$

which leads to

23 $$p=\frac{2fQ_{1}Q_{2}}{\left(fQ_{1}+Q_{2}\right)}d$$

and

24 $$Q_{\mathrm{net}}=Q_{2}-fQ_{1}$$

**Scheme 4:**
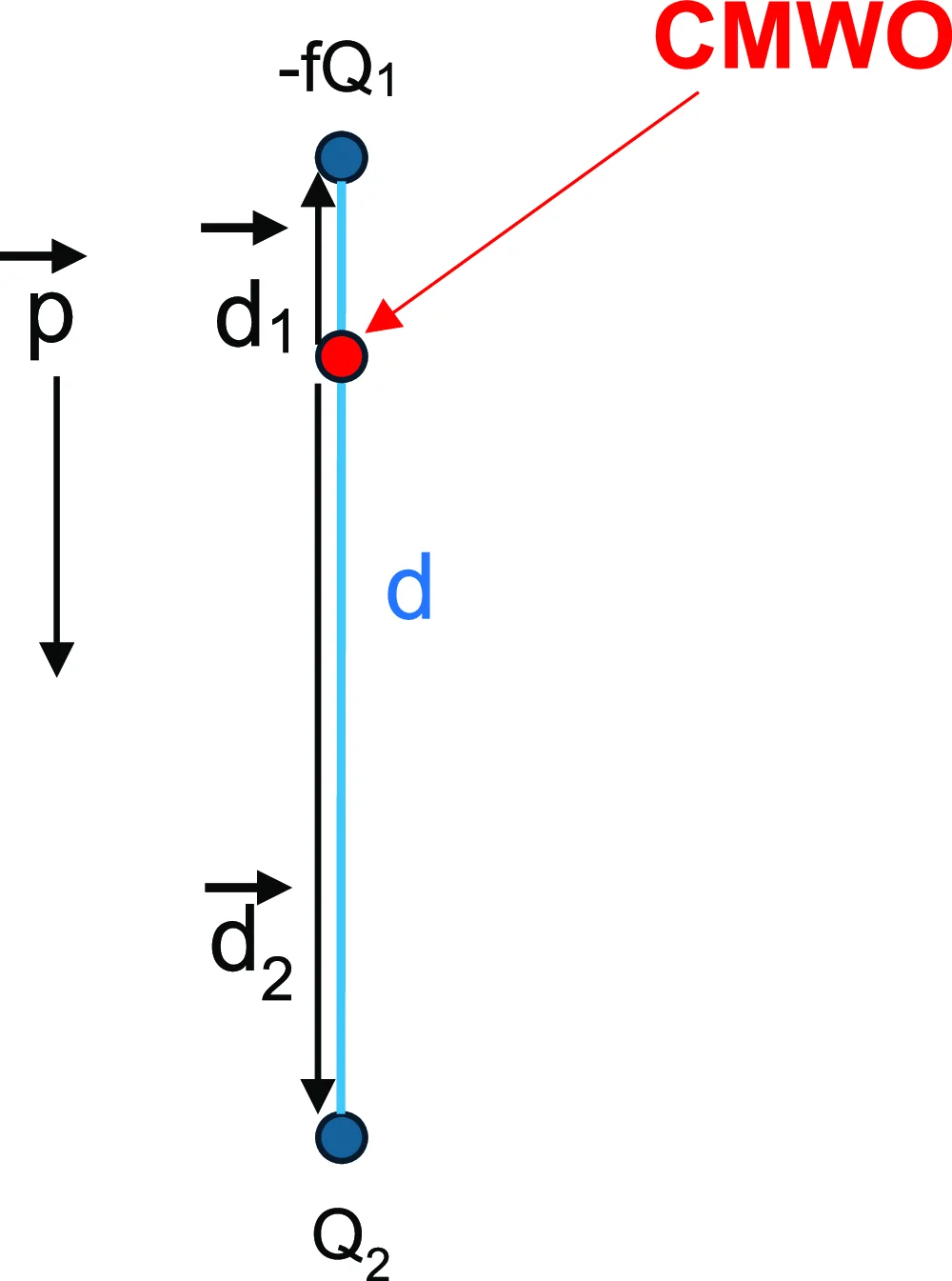
Charge-Magnitude-Weighted-Origin Gauge Geometry for a Single SG or SG Micelle, Representing the Negative Patch by *-Q*_1_ and the Positive uPatch by *Q*_2_ [Fn s4fn1]

a
*Q*_1_ and *Q*_2_ are Both Positive.

Equation 31 captures the limits (i) *p* = 0 when *f* = 0 (no net negative charge. Compare this to the geometrical center value of *p* = *Q*_2_ *d*/2), and (ii) *p = Qd* when *Q*_1_ = *Q*_2_ *= Q*, *f* = 1 (agrees with geometrical center value)

Letting

25 $$\alpha =\frac{Q_{1}}{Q_{2}}$$

gives single-charge (*Q*_2_) expressions for *Q*_net_ and *p*

26a $$Q_{\mathrm{net}}=Q_{2}\left(1-\alpha f\right)$$

26b $$p=\frac{2\alpha fQ_{2}}{\left(1+\alpha f\right)}d$$

The ratio of *p* for the CMWO, *p*_CMWO_ and the geometrical center origin (*d*/2) *p*_GC_ is

26c $$\frac{p_{\mathrm{CMWO}}}{p_{\mathrm{GC}}}=\frac{4\alpha }{{\left(1+\alpha \right)}^{2}}$$

For which this ratio is in pairs $\left(\alpha ,\frac{p_{\mathrm{CMWO}}}{p_{\mathrm{GC}}}\right)$ ; e.g., (0.1, 0.18), (0.5, 0.89), (2,0.89), (4,0.64), (10,0.33)

This shows that the, *p*_CMWO_ is close to *p*_GC_ when *Q*_1_ and *Q*_2_ are not far apart, but p is significantly modulated downward when they differ significantly. Equation 36 is also symmetric under α → 1/α, so that over- or under-charging modulates *p* downward equally.

Linking *p* and *Q*_net_ via eqs 31 and 32 gives

27a $$\frac{Q_{\mathrm{net}}}{p}=\frac{Q_{2}^{2}-f^{2}Q_{1}^{2}}{2{\mathrm{fQ}}_{1}Q_{2}d}$$

For NaCl, *f* = 1, reduces eq 37 to

27b $$\frac{Q_{\mathrm{net}}}{p}=\frac{Q_{2}^{2}-Q_{1}^{2}}{2Q_{1}Q_{2}d}$$

and eq 37 can be written

27c $$\frac{Q_{\mathrm{net}}}{p}=\frac{1-{\alpha^{2}f}^{2}}{2\alpha \mathrm{fd}}$$

Referring to eqs 21,22, the prefactors cancel upon taking the ratio of [I]_t_ for Gdn/NaCl, leaving the scaling law, ${\left(\frac{z_{\mathrm{net}}}{n_{\mathrm{net}}}\right)}^{2}$ The ratio *B*, of the square roots of the experimentally determined ionic strength thresholds [Gdn]_t_, and [NaCl]_t_, can be directly related to this scaling law, such that

28a $$B\equiv \sqrt{\frac{{\left[\mathrm{Gd}\right]}_{t}}{{\left[\mathrm{NaCl}\right]}_{t}}}=\frac{{\left(\frac{Q_{\mathrm{net}}}{p}\right)}_{{\left[\mathrm{Gd}\right]}_{t}}}{{\left(\frac{Q_{\mathrm{net}}}{p}\right)}_{{[\mathrm{NaCl}]}_{t}}}$$

In terms of *f*

28b $$B=\frac{1-\alpha^{2}f^{2}}{f\left(1-\alpha^{2}\right)}$$

Note that if *Q*_1_ = *Q*_2_ *= Q* – i.e., at the isoelectric point of the GLPA-- then *Q*_net_ = 0 for the GLPA in NaCl, since *f* = 1. In this case there is no repulsive term, but there is an attractive dipole moment term, since *p = Qd* in this case. Hence [I]_t,NaCl_=0, indicating that the system will spontaneously aggregate when *Q*_*1*_ *=Q*_*2*_ and *f* = 1, even with no added electrolyte, assuming there are no steric or other effects creating a residual energy barrier. On the other hand, for *Q*_*1*_ *= Q*_*2*_ and the GLPA in Gdn, *f* < 1, and so *Q*_net_ ≠ 0. Thus, there is a finite, nonzero [I]_t,Gdn_, and so *B* in eq 41 diverges to infinity. This divergence is quite physical, since $\frac{{\left[I\right]}_{t,\mathrm{Gd}}}{{\left[I\right]}_{t,\mathrm{NaCl}}}=\frac{\neq 0}{0}$ .

Using eq 41 with *f* for Gdn and *f* = 1 for NaCl leads to the quadratic equation

29a $$\alpha^{2}f^{2}+B\left(1-\alpha^{2}\right)f-1=0$$

with solution

29b $$f=\frac{B\left(\alpha^{2}-1\right)+\sqrt{B^{2}{\left(1-\alpha^{2}\right)}^{2}+4\alpha^{2}}}{2\alpha^{2}}$$

where *B* is the $\sqrt{\frac{{\left[\mathrm{Gd}\right]}_{t}}{{\left[\mathrm{NaCl}\right]}_{t}}}$ term in eq 40. Considerations on estimating α are next.

### Absence of Experimental Data on SG Charge vs pH. Use of Computed Values

In order to take advantage of the above development it is necessary to know *Q*_1_ and *Q*_2_ vs pH, as well as *d* and *K*_ap_. Unfortunately, the authors could find no reference giving experimental data for either net charge or number of positive and negative charges vs pH for GLPA micelles. Hence, while the above approximate model is quantitative, within the stated approximations, the values of *Q*_*1*_, *Q*_*2*_, *K*_ap_, and *d* can only be estimated at this time.

As a starting point, computations of *Q*_1_ and *Q*_2_ were made by simply using the Henderson–Hasselbach equations (HH) for the specified amino acid sequences in semaglutide and liraglutide. This approach is rudimentary in that it does not take into account how the effective p*K*_a_’s of the acid/base groups in a polymer shift with respect to their value for isolated acids/bases,^50–52^ including very large p*K*_a_ shifts for amino acids in different proteins.^53^ These values are shown for the SG and LG monomers in the *Q*_1,HH_/e and *Q*_2,HH_/e (Henderson-Hasselbach) columns of Table 2. SG has a lysine that remains protonated, giving it more positive charge at any pH than LG, because the lysine on LG is capped by the fatty acid chain and has no positive charge. This simple difference leads to substantial differences in dipole moment and aggregation behavior. This is seen in Table 2 where LG requires more NaCl (and Gdn) for aggregation than SG.

Recently, the effects of pH and various electrolytes on semaglutide aggregation, size distributions and zeta potential have been published, along with their effects on filtration.^54^

### Estimation of *f*, *K*_ap_, and *p*_monomer_ from [Gdn]_t_, [NaCl]_t_, and Q_1,HH_ and Q_2/HH_

Using the HH values for charge, the model allows estimation of the monomer dipole moment *p*_monomer_, and the fraction, *f*, of Gdn^+^ bound to the negative amino acids, and associated apparent binding constants *K*_ap_. Table 2 shows *f* and *K*_ap_ for SG at three pH’s and LG at two pH’s. α (=*Q*_1,HH_/*Q*_2,HH_) is used in eq 43 to obtain *f*. With *f* thus found, *K*_ap_ is obtained from eq 27. Notice that the use of α means that *Q*_1_ and *Q*_2_ apply equally to a monomer with these charges, and to a micelle or aggregate with any other pair of *Q*_1_ and *Q*_2_. Again, *Q*_1_ and *Q*_2_ for the monomers are not experimentally known, and rely on the free amino acid p*K*_a_’s. How *Q*_1_ and *Q*_2_ in the micelle are related to the monomeric values is unknown, as is also the net dipole moment of a micelle built from monomer dipoles *p*. While *f* and *K*_ap_ depend only on α and the experimentally measured B, the values of *Q*_net_ and *p*_net_ depend on *Q*_2_ (or on *Q*_1_ if the reciprocal of α is used)

30 $$Q_{\mathrm{net}}=Q_{2}\left(1-\alpha f\right)$$

and the monomeric dipole moments in Table 2 are computed by

31 $$p_{\mathrm{monomer}}=\frac{2f\alpha Q_{2}d}{1+\alpha f}$$

with *f* = 1 and *d*∼1 nm was used as a plausible estimate. The estimates of *p*_monomer_ in Table 2 range from 115 D down to 15 D. For reference, the permanent dipole moment of water is 1.85D, whereas those of globular proteins can exceed 1000 D. The net dipole moment of the micelle cannot easily be deduced from the data. The ratio of the *U*_m‑m_ term (∼*Q*_net_^2^) to the sum of the *U*_m‑d_ and *U*_d‑d_ in eq 12 depends on the values of *r* and κ chosen and the multiples of *p*_monomer_ assumed to constitute the net micelle dipole moment *p*_*net*_ (e.g., 8p_monomer_).

While the values of *p*_monomer_ in Table 2 are only estimates, they suggest that SG has a significantly larger dipole moment than LG at the pH values used. This is because the putative positive charge, *Q*_2_, is less at each pH for LG than for SG, while the negative charges, *Q*_1_, are comparable. *Q*_net_ has greater magnitude for LG at each pH than SG. This means that there is a larger electrostatic barrier, *U*_m‑m_, for LG than SG, while the smaller LG dipoles provide less attraction, so that *Q*_net_ must be screened more heavily for aggregation to occur for LG than for SG. In fact at pH 8.15 [NaCl]_p_= 2.78 M for LG while at a comparable pH (8.6) [NaCl]_t_= 1.50 M for SG (and [NaCl]_t_ would be even less than 1.50 M at pH 8.15). By the same token, [Gdn]_t_ is much higher for LG than for SG, also due to the higher *Q*_net_ and weaker dipoles of LG. The attractive *U*_m‑d_ and *U*_d‑d_ both weaken as *p*_net_ decreases. At the extremes of pH *p*_net_ = 0, since *Q*_2_ → 0 at very high pH and Q_1_ → 0 at very low pH.

The former interpretation is consistent within the two GLPA molecules, SG and LG. As pH increases *Q*_net_ increases in magnitude and the dipole weakens in each case, leading to higher threshold values at higher pH for both Gdn and NaCl in each case.

While beyond the scope of this work, the dipole moments of the GLPA could conceivably be measured by Dielectric Relaxation Spectroscopy^55,56^ or by Electric Birefringence Spectroscopy.^57,58^

Figure 11 shows the [NaCl]_t_ measured by light scattering vs *z*_net_/*n*_*net*_ [=(*Q*_net_/*p*)*­(D/e)] for SG, taken from the data in Table 2, including the experimentally measured aggregation thresholds. While the data are sparse and the interpretation rests on the approximations made, the behavior is parabolic, predicted by the parabolas of eqs 21,22, with a prefactor of 588, which is a bit more than double the value of eq 22. There is also an offset, which is not predicted and is likely an artifact of the data and approximations. Meanwhile, the pH increases exponentially with *z*_net_/*n* (pH on right-hand axis is logarithmic).

**Figure 11:**
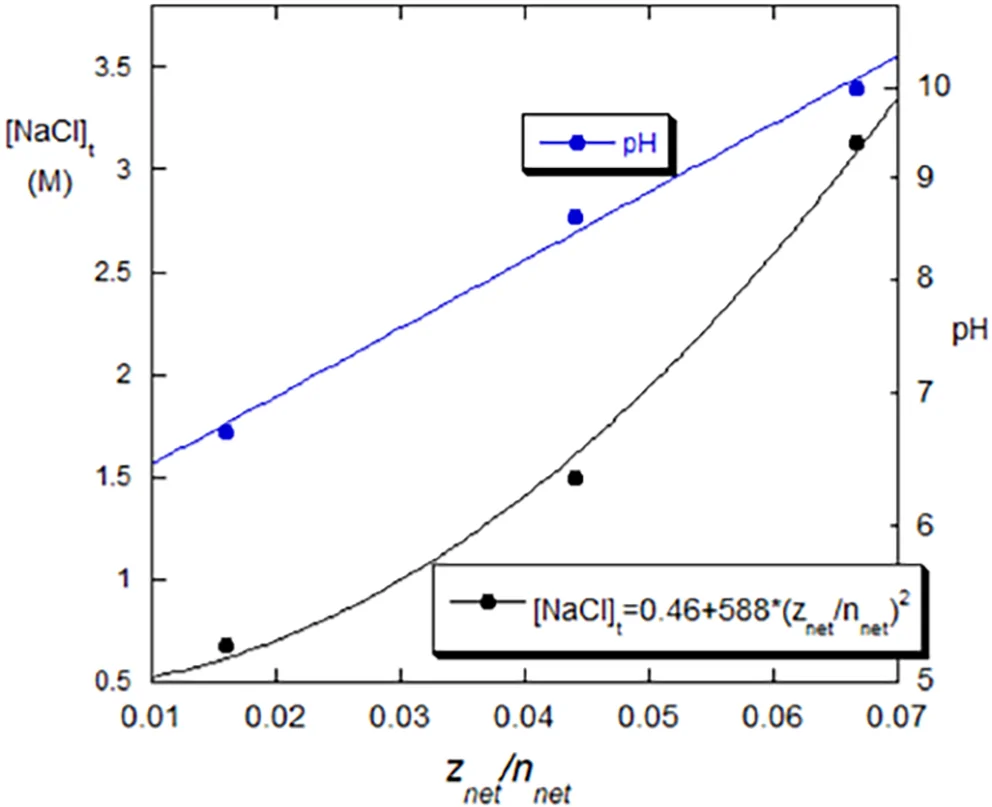
Parabolic dependence of [NaCl]_t_ on *z*_net_/*n*, predicted by eqs 21a,22. The pH is exponential in *z*_net_/*n*.

### Temperature Behavior

Figures 7a–c and 8a–c show the behavior of the GLPA under temperature ramps and the time trajectory at several fixed temperatures, respectively. All three GLPAs initially lose mass under both temperature ramps and at fixed temperatures. This likely corresponds to the loss of one or more monomers in the GLPA micelle, or the breaking up of micelles into smaller ones. While the micellization is driven at least in part by the classical hydrophobic effect, due to the long chain fatty acid bonded to the GLPA, this effect strengthens as T increases, which could lead to increasing the number of monomers in the micelle. This is in the opposite direction of losing a monomer with increasing T, which could signal either loss of H-bonds, if these are involved in micellization, or decreased π–π stacking, if any, or a change in net charge, due to the p*K*_a_ of amino acids decreasing with increasing T.

Unlike typical globular proteins that irreversibly aggregate upon heating, these GLPA show reversible monomer loss, or micelle fragmentation, and slow restructuring, clearly separating the behavior of these oligopeptides from the globular proteins.

Whatever the mechanism of the monomer loss as T increases, it is a slow process, with characteristic times at least 100 s for dissociation, and at least 10,000 s for reassociation. This implies some very elaborate changes in the micelle structure, some sort of collective structural relaxation, especially involving the interaction of the fatty acids, as opposed to a virtually instantaneous change in charge on any of the groups; e.g., if repulsion increases due to further amino acid deprotonation, it takes time for the elaborate architecture of a > 20,000 g/mol structure to conformationally react to the change in charge state. Similarly, the fact that the original state is regained after an even longer time for SG and LG suggests that there is likely significant conformational restructuring to allow this.

### Conjecture on Aggregate Structure

GLPA aggregates are known to form a wide variety of structures, including amorphous^59^ types and amyloid fibrils.^60,61^ Native GLP-1 can also form amyloid fibrils.^62,63^ It is conjectured here that the alignment of dipoles which has made a compelling model for GLPA aggregation could lead to chains of aligned dipoles of the micelles, yielding a fibril type structure. Interacting dipoles can form a variety of structures.^64^

## Supporting Evidence for the Electrostatic Hypothesis

The central hypothesis of this work is that asymmetric electrostatics largely control the aggregation of the GLPA micelles. The evidence has been laid out in detail, and is summarized here.

- The formation of the GLPA micelles is driven by the classical hydrophobic effect, while the colloidal aggregation of the micelles is largely controlled by monopole and dipole electrostatics.
- Increasing ionic strength screens the electrostatic repulsion between micelles, progressively lowering the Coulomb barrier to association. When this barrier falls to only a few *k*_B_ *T*, thermal fluctuations allow micelles to approach closely enough for short-range attractive interactions to stabilize aggregates.
- Hydrophobic interactions are short-range, and only weakly dependent on ionic strength, and do not naturally produce sharp ionic-strength thresholds. In contrast, electrostatic stabilization of colloidal particles naturally produces threshold behavior: repulsive Coulomb interactions create an energy barrier to association, and increasing ionic strength progressively screens this repulsion. When screening reduces the barrier to only a few *k*_B_ *T*, thermal fluctuations permit micelles to approach to distances where short-range attractions can stabilize aggregates. The observed thresholds in NaCl and guanidinium chloride are therefore consistent with aggregation controlled by electrostatic barrier collapse.
- There is no aggregation of GLPA micelles caused by increasing temperature (Figure 7a). Globular proteins, whose aggregation is driven largely by the hydrophobic effect, unfold and aggregate as T increases. For the GLPA micelles the only effect of T is to reversibly reduce the number of monomers in the micelles. There is no detectable unfolding and aggregation, suggesting a minimal effect of hydrophobicity, if any.
- When dialyzing GLPA micelles against Gdn, colloidal aggregation begins at an order of magnitude lower than with NaCl. This is caused by the charge neutralization of negatively charged amino acids by Gdn^+^, which suppresses m-m repulsion, leading to early aggregation. If hydrophobic effects were involved, Gdn would suppress it, and hence there would be no aggregation. The opposite is found.
- Changing pH has a very strong effect on aggregation. This is because pH changes the GLPA charge state, and hence both the net charge and the charges constituting the dipoles. The hydrophobic effect is not normally dramatically altered by changes in pH.
- The aggregation thresholds and peaks generally occur below 1 M NaCl and Gdn, a regime in which Debye–Hueckel electrostatics should be a reasonable approximation.
- SG has more positive charge at any pH than LG, because the lysine on LG is capped by the fatty acid chain and has no positive charge.
- The electrostatic model proposed demonstrates that there are repulsive, stabilizing forces due to monopole-monopole repulsion, but that there are strong, orientationally dependent, attractive, destabilizing monopole-dipole and dipole–dipole forces
- The model makes specific predictions about the instability threshold. Equations 21,22, predict the parabolic dependence of the aggregation threshold on (*Q*_net_/*p*_net_)^2^ seen in Figure 11, and the strikingly lowered threshold for Gdn+ due to Gdn+ adsorption onto negatively charged amino acids, with consequent lowering of *Q*_net_.
- A first order estimate (Henderson-Hasselbach) finds plausible values for positive and negative charges on SG and LG, as well as associated dipole moments. The estimated dipole moments in Table 1 for GLPA monomers are quite high.

## Limitations of the Model

The present model is intentionally coarse-grained and aims to determine whether anisotropic electrostatic interactions can account for the principal experimental trends. The model treats GLP-1 analog monomers, micelles, and aggregates as effective charged entities possessing net point charge and dipole moments, without explicitly incorporating detailed morphology, charge-patch geometry, hydrogen bonding, hydrophobic interactions, π-π interactions, or higher order multipoles. The model should be regarded as a first-order physical framework rather than a complete microscopic description. It is expected that the model should be applicable to other peptide systems, but will depend on their specific charge distributions, morphologies, and solution conditions, and may also require including the effects ignored here.

## Summary

At equilibrium, the GLPA form micelle-like associations, “micelles”, with 5–8 monomers, depending on the specific GLPA. GLPA contain a fatty acid residue that provides the hydrophobicity for the micellization; liraglutide has a C16 palmitic acid chain, while semaglutide has a C18 stearic diacid. In the analysis, the GLPA micelle is taken as the basic unit which aggregates under NaCl and Gdn. At 0.0035 g/mL, where most of the dialysis was performed, the average distance between GLPA micelles of *M* = 30,000 is about 24 nm.

Under dialysis with a simple electrolyte (NaCl) and a chaotropic salt (Guanidine-HCl) SG has an aggregation threshold concentration, termed [NaCl]_t_ and [Gdn]_t_, leading to a peak and a subsequent window of strong aggregation for higher [NaCl] and [Gdn]. LG also showed peaks, but without an abrupt threshold. The data clearly support the notion that the dialysis behavior, including the aggregation threshold and peak, is due mostly to multipolar electrostatic interactions, with possible smaller contributions from H-bonds, π–π stacking, and the classical hydrophobic effect. This is quite different from RNA aggregation (dominated by π–π stacking), and from aggregation of unfolded globular proteins (dominated by the classical hydrophobic effect when the protein unfolds).

The electrostatic origin of aggregation is qualitatively plausible, because the GLPA are peptides with positive and negative charge patches that give them a net charge at any pH (except at the isoelectric point, about 4.9 for liraglutide and 5.4 for semaglutide), but also always a dipole moment, even at the IP. The data indicate there is a net dipole moment in the micelles; i.e., the monomer dipoles do not cancel out in the micelle. This is quantitatively borne out by the electrostatic model, which is based on the repulsive monopole-monopole (m-m) interaction energy, *U*_m‑m_ and net attractive monopole-dipole (m-d), *U*_m‑d_ and dipole–dipole (d-d), *U*_d‑d_, interaction energies. The GLPA also have quadrupole and higher moments which might contribute relatively small, very short-range attractive interactions, but they are ignored here.

The electrostatic model introduced here is independent of GLPA morphology; it merely requires that any GLPA entity have a net charge and net dipole moment, both treated as pointlike, and hence implies an electrostatic universality among GLPA morphologies. Details of the net charge distribution, dipole moment and distances involved will, of course, modulate these parameters, and hence the quantitative particulars of each morphological case. In addition to interpreting the data trends, the model yields estimates of GLPA dipole moments, and fractional binding of the Gdn^+^ ion to negative amino acids, and associated binding constants.

When dialyzing with NaCl the net charge and dipole moment at a given pH remain the same, so it is screening of the net charge that allows two GLPA to get close enough together to experience the attractive *U*_m‑d_ and *U*_d‑d_ potentials and to aggregate. When pH increases the negative charge increases and the positive charge decreases, leading to a higher net negative charge, while the dipole moment decreases. The increase of the former increases the repulsive *U*_m‑m_, while a decrease of the latter decreases the strength of the attractive *U*_m‑d_ and *U*_d‑d_ potentials. These effects lead to requiring more NaCl to bring two GLPA close enough to each other’s weakened attractive potential.

In the case of dialysis with Gdn, a key phenomenon is the binding of the Gdn cationic portion, Gdn^+^, to negatively charged amino acid residues. This neutralizes part of the GLPA negative charge, describable phenomenologically via a Langmuir binding curve, and hence reduces the net negative charge of the GLPA (above the IP), and also reduces the dipole moment. GLPA at any given pH and [Gdn], can have either a sharp threshold or broad, peaked onset, as seen in Table 2. The differences between the threshold or peak are dramatically lower for Gdn than for NaCl. Threshold and peak values for NaCl and Gdn cover a broad range from 0.077 M to 3.2M.

Because GLPA have net charge away from their IP, their dipole moment depends on where the origin of the dipole is placed between the two charges, a negative *Q*_1_ at a distance *d* from a positive charge *Q*_2_. To reduce this dependence on an arbitrary origin of the dipole (such as the midpoint connecting *Q*_1_ and *Q*_2_), the charge-magnitude-weighted origin (CMWO) is used as a gauge, which depends on the relative charges of *Q*_1_ and *Q*_2_, and corrects for overestimating the dipole moment when there is a large difference between *Q*_1_ and *Q*_2_.

The order of thermal stability (lowest to highest stability) is LG< SG. SG gives perfect reversibility for up and down T-ramps from 25 to 60 °C, while LG is close to fully reversible. The T-ramps show that all the GLPA micelles here lose monomer as T increases, and/or the micelles dissociate into smaller ones. Because the classical hydrophobic effect normally strengthens with increasing T, up to about 50 °C, this loss of monomer suggests that the classical hydrophobic effect due to the fatty acid side chains on the GLPA monomers is only partly responsible for the formation of the micelle. The attractive electrostatic energies *U*_m‑d_ and *U*_d‑d_ may also play a prominent role in stabilizing the micelle, while the effect of H-bonds and π–π stacking may be much less. The possibility that the particularities of the GLPA here push the temperature weakening of the hydrophobic effect down to as much as 30 °C cannot be excluded.

In the current physicochemical R&D space for GLPA, also including tirzepatide, survodutide, and others, there are many chemical modifications being explored and optimized. These include amino acid substitutions, altering the fatty acid chain lengths, types, branching, number of chains, and linker type. Additionally, formulations with varying compositions of buffer, phenol, propylene glycol and other excipients are under active research.

For stability against aggregation, it is best to maximize net charge and minimize the dipole moment. For example, a single dialysis scan against NaCl or other electrolyte will quickly show the aggregation threshold for the electrolyte and allow a quick diagnostic comparison among different candidate GLPA’s and formulations. A further dialysis scan with guanidine-HCl can reveal whether cation binding to amino acid residues is occurring. Similarly, whether other cations may bind to GLPA and hence reduce their stability can also be quickly determined. In such cases the fraction of sites occupied and binding constants can be assessed. The ability of the dialysis method to also monitor the effects of continuously changing pH can map out the ranges of highest stability.

The dialysis approach, and subsequent modeling, should help to better understand GLPA stability under different chemistries and formulations, and also to better understand the stability of other oligopeptides such as Conotoxins, substance P antagonists, Thymosin beta4 fragments, GnRH analogs, somatostatin analogs, α MSH analogs, and others.

## References

[1] Muscogiuri G., DeFronzo R. A., Gastaldelli A., Holst J. J. (2017). Glucagon-like Peptide-1
and the Central/Peripheral Nervous System: Crosstalk in Diabetes. Trends Endocrinol. Metab..

[2] Müller T., Finan B., Bloom S. R., D’Alessio D., Drucker D. J., Flatt P. R., Fritsche A., Gribble F., Grill H. J., Habener J. F., Holst J. J., Langhans W., Meier J. J., Nauck M. A., Perez-Tilve D., Pocai A., Reimann F., Sandoval D. A., Schwartz T. W., Seeley R. J., Stemmer K., Tang-Christensen M., Woods S. C., DiMarchi R. D., Tschöp M. H. (2019). Glucagon-like
peptide 1 (GLP-1). Mol. Metab..

[3] Holst J. J. (2019). From the
Incretin Concept and the Discovery of GLP-1 to Today’s Diabetes
Therapy. Front. Endocrinol..

[4] Seufert J., Gallwitz B. (2014). The extra-pancreatic
effects of GLP-1 receptor agonists:
a focus on the cardiovascular, gastrointestinal and central nervous
systems. Diabetes, Obes. Metab..

[5] Singh G., Krauthamer M., Bjalme-Evans M. (2022). Wegovy (semaglutide): a new weight
loss drug for chronic weight management. J.
Invest. Med..

[6] Blundell J., Finlayson G., Axelsen M., Flint A., Gibbons C., Kvist T., Hjerpsted J. B. (2017). Effects of once-weekly semaglutide
on appetite, energy intake, control of eating, food preference and
body weight in subjects with obesity. Diabetes,
Obes. Metab..

[7] Latif, W. ; Lambrinos, K. J. ; Patel, P. ; Rodriguez, R. Compare and Contrast the Glucagon-Like Peptide-1 Receptor Agonists (GLP1RAs); StatPearls: Treasure Island (FL), 2025. 34283517

[8] Wang Y., Lomakin A., Kanai S., Alex R., Benedek G. B. (2015). Transformation
of oligomers of lipidated peptide induced by change in pH. Mol. Pharmaceutics.

[9] Staby A., Steensgaard D. B., Haselmann K. F., Marino J. S., Bartholdy C., Videbæk N., Schelde O., Bosch-Traberg H., Spang L. T., Asgreen D. J. (2020). Influence of Production Process and
Scale on Quality of Polypeptide Drugs: a Case Study on GLP-1 Analogs. Pharm. Res..

[10] Hamley I. W., de Mello L. R., Castelletto V., Zinn T., Cowieson N., Seitsonen J., Bizien T. (2025). Semaglutide Aggregates into Oligomeric
Micelles and Short Fibrils in Aqueous Solution. Biomacromolecules.

[11] Bothe J. R., Andrews A., Smith K. J., Joyce L. A., Krishnamachari Y., Kashi S. (2019). Peptide Oligomerization Memory Effects
and Their Impact on the Physical
Stability of the GLP-1 Agonist Liraglutide. Mol. Pharmaceutics.

[12] Castelletto V., de Mello L. R., Seitsonen J., Hamley I. W. (2026). Fibrillar and Micellar
Aggregation of Semaglutide and Formation of a Chiral-Imprinted Glass. Biomacromolecules.

[13] Malgave A., Akbar S., Joseph A., Aishwarya D., Peraman R., Malayandi R. (2025). Effect of pH, buffers, molarity,
and temperature on solution state degradation of semaglutide using
LC-HRMS: A preformulation protocol for peptide drug delivery. Eur. J. Pharm. Biopharm..

[14] Zapadka K. L., Becher F. J., Dos Santos A. L. G., Jackson S. E. (2017). Factors affecting
the physical stability (aggregation) of peptide therapeutics. Interface Focus.

[15] Marek P. J., Patsalo V., Green D. F., Raleigh D. P. (2012). Ionic Strength Effects
on Amyloid Formation by Amylin Are a Complicated Interplay between
Debye Screening, Ion Selectivity, and Hofmeister Effects. Biochemistry.

[16] Giannetti M., Palleschi A., Ricciardi B., Venanzi M. (2023). A Spectroscopic and
Molecular Dynamics Study on the Aggregation Properties of a Lipopeptide
Analogue of Liraglutide, a Therapeutic Peptide against Diabetes Type
2. Molecules.

[17] Brichtova E. P., Santos A. L. G. D., Jackson S. E. (2025). Observation of unique
stable nano-assemblies
of a lipidated glucagon-like peptide 1 analogue. Soft Matter.

[18] Striolo A., Bratko D., Wu J. Z., Elvassore N., Blanch H. W., Prausnitz J. M. (2002). Forces
between aqueous nonuniformly
charged colloids from molecular simulation. J. Chem. Phys..

[19] Brunsteiner M., Flock M., Nidetzky B. (2013). Structure Based Descriptors for the
Estimation of Colloidal Interactions and Protein Aggregation Propensities. PLoS One.

[20] Lee H. M., Kim Y. W., Go E. M., Revadekar C., Choi K. H., Cho Y., Kwak S. K., Park B. J. (2023). Direct
measurements of the colloidal Debye force. Nat.
Commun..

[21] Gnidovec A., Locatelli E., Čopar S., Božič A., Bianchi E. (2025). Anisotropic DLVO-like interaction for charge patchiness
in colloids and proteins. Nat. Commun..

[22] Bianchi E., van Oostrum P. D. J., Likos C. N., Kahl G. (2017). Inverse patchy colloids:
Synthesis, modeling and self-organization. Curr.
Opin. Colloid Interface Sci..

[23] Li W., Persson B. A., Morin M., Behrens M. A., Lund M., Zackrisson Oskolkova M. (2015). Charge-Induced Patchy Attractions between Proteins. J. Phys. Chem. B.

[24] Jarand C. W., McLeod M. J., Reed W. F. (2024). Dialysis Monitoring of Ionic Strength
and Denaturant Effects, and Their Reversibility, for Various Classes
of Macromolecules. Biomacromolecules.

[25] Jarand, A. ; Jarand, C. ; Reed, W. Miniature conductivity and pH sensors using ion selective field effect transistors applied in cuvette-based dialysis spectroscopy - IOPscience. https://iopscience.iop.org/article/10.1088/1361-6501/ae34d4.

[26] Farkas N., Kramar J. A. (2021). Dynamic light scattering
distributions by any means. J. Nanopart. Res..

[27] Epstein C. L., Schotland J. (2008). The Bad Truth
about Laplace’s Transform. SIAM Rev..

[28] Franks K., Kestens V., Braun A., Roebben G., Linsinger T. P. J. (2019). Non-equivalence
of different evaluation algorithms to derive mean particle size from
dynamic light scattering data. J. Nanopart.
Res..

[29] Dynamic Light Scattering: The Method and Some Applications; Brown, W. , Ed.; Oxford University Press:: Oxford, NY, 1993.

[30] Venanzi M., Savioli M., Cimino R., Gatto E., Palleschi A., Ripani G., Cicero D., Placidi E., Orvieto F., Bianchi E. (2020). A spectroscopic and molecular dynamics study on the
aggregation process of a long-acting lipidated therapeutic peptide:
the case of semaglutide. Soft Matter.

[31] Frederiksen T. M., Sønderby P., Ryberg L. A., Harris P., Bukrinski J. T., Scharff-Poulsen A. M., Elf-Lind M. N., Peters G. H. (2015). Oligomerization
of a Glucagon-like Peptide 1 Analog: Bridging Experiment and Simulations. Biophys. J..

[32] Hamley I. W., de Mello L. R., Castelletto V., Zinn T., Cowieson N., Seitsonen J., Bizien T. (2025). Semaglutide Aggregates Into Oligomeric
Micelles and Short Fibrils in Aqueous Solution. Biomacromolecules.

[33] Hammouda B. (2013). Temperature
Effect on the Nanostructure of SDS Micelles in Water. J. Res. Natl. Inst. Stand. Technol..

[34] Chun B. J., Choi J. I., Jang S. S. (2015). Molecular
dynamics simulation study
of sodium dodecyl sulfate micelle: Water penetration and sodium dodecyl
sulfate dissociation. Colloids Surf., A.

[35] Tanford, C. The Hydrophobic Effect: Formation of Micelles and Biological Membranes; Wiley: New York, 1973.

[36] Israelachvilli, J. Intermolecular and Surface Forces.

[37] Božič A. L., Podgornik R. (2017). pH Dependence of Charge Multipole Moments in Proteins. Biophys. J..

[38] Jarand C. W., Deng Z., Brader M., Reed W. (2026). Consequences
of mRNA
Secondary Structure on Stability against both Hydrolysis and Aggregation:
The Role of Electrostatic, π–π Stacking, and Thermal
Effects. ACS Omega.

[39] Hoerschinger V. J., Waibl F., Pomarici N. D., Loeffler J. R., Deane C. M., Georges G., Kettenberger H., Fernández-Quintero M. L., Liedl K. R. (2023). PEP-Patch: Electrostatics
in Protein-Protein Recognition,
Specificity, and Antibody Developability. J.
Chem. Inf. Model..

[40] Di
Savino A., Foerster J. M., Ullmann G. M., Ubbink M. (2021). The Charge
Distribution on a Protein Surface Determines Whether Productive or
Futile Encounter Complexes Are Formed. Biochemistry.

[41] Keith A. D., Brichtová E. P., Barber J. G., Wales D. J., Jackson S. E., Röder K. (2024). Energy Landscapes
and Structural Ensembles of Glucagon-like
Peptide-1 Monomers. J. Phys. Chem. B.

[42] Brichtová E. P., Edu I. A., Li X., Becher F., dos Santos A. L. G., Jackson S. E. (2025). Effect of Lipidation
on the Structure, Oligomerization,
and Aggregation of Glucagon-like Peptide 1. Bioconjugate Chem..

[43] Hamley I. W., Castelletto V. (2023). Small-angle
scattering techniques for peptide and peptide
hybrid nanostructures and peptide-based biomaterials. Adv. Colloid Interface Sci..

[44] Hutchinson J. A., Burholt S., Hamley I. W., Lundback A.-K., Uddin S., Dos Santos A. G., Reza M., Seitsonen J., Ruokolainen J. (2018). The Effect
of Lipidation on the Self-Assembly of the
Gut-Derived Peptide Hormone PYY3–36. Bioconjugate Chem..

[45] Ramirez R., Kjellander R. (2006). Effective
multipoles and Yukawa electrostatics in dressed
molecule theory. J. Chem. Phys..

[46] Tsonchev S., Schatz G. C., Ratner M. A. (2004). Screened
multipole electrostatic
interactions at the Debye–Hückel level. Chem. Phys. Lett..

[47] Ahmad S., Sarai A. (2011). Analysis of electric moments of RNA-binding
proteins: implications
for mechanism and prediction. BMC Struct. Biol..

[48] Levina E. O., Lokshin B. V., Mai B. D., Vener M. V. (2016). Spectral features
of guanidinium-carboxylate salt bridges. The combined ATR-IR and theoretical
studies of aqueous solution of guanidinium acetate. Chem. Phys. Lett..

[49] Rozanska X., Chipot C. (2000). Modeling ion–ion interaction
in proteins: A
molecular dynamics free energy calculation of the guanidinium-acetate
association. J. Chem. Phys..

[50] Katchalsky A., Shavit N., Eisenberg H. (1954). Dissociation
of weak polymeric acids
and bases. J. Polym. Sci..

[51] Pineda S. P., Staňo R., Murmiliuk A., Blanco P. M., Montes P., Tošner Z., Groborz O., Pánek J., Hrubý M., Štěpánek M., Košovan P. (2024). Charge Regulation
Triggers Condensation of Short Oligopeptides
to Polyelectrolytes. JACS Au.

[52] Reed C. E., Reed W. F. (1992). Monte Carlo study
of titration of linear polyelectrolytes. J.
Chem. Phys..

[53] Pahari S., Sun L., Alexov E. (2019). PKAD: a database
of experimentally measured pKa values
of ionizable groups in proteins. Database.

[54] Duan L., Yang Y., Wu H., Ding X., Jiang M. (2026). Effects of
pH and Salts on the Aggregation State of Semaglutide and Membrane
Filtration Performance. Separations.

[55] Williams G. (1994). Dielectric
relaxation spectroscopy of polymers revealing dynamics in isotropic
and anisotropic stationary systems and changes in molecular mobility
in non-stationary systems. Polymer.

[56] Yamamoto W. (2013). Dielectric
relaxation strength and magnitude of dipole moment of poly­(vinyl pyrrolidone)­in
polar solutions. J. Mol. Liq..

[57] Nagai K., Ishikawa T. (1965). Internal Rotation and Kerr Effect in Polymer Molecules. J. Chem. Phys..

[58] Bellini T., Degiorgio V., Mantegazza F. (1998). The electric birefringence of polyelectrolytes:
an electrokinetic approach. Colloids Surf.,
A.

[59] Trainor K., Broom A., Meiering E. M. (2017). Exploring
the relationships between
protein sequence, structure and solubility. Curr. Opin. Struct. Biol..

[60] Riek R., Eisenberg D. S. (2016). The activities of amyloids from a
structural perspective. Nature.

[61] Zapadka K. L., Becher F. J., Uddin S., Varley P. G., Bishop S., Dos Santos A. L. G., Jackson S. E. (2016). A pH-Induced Switch in Human Glucagon-like
Peptide-1 Aggregation Kinetics. J. Am. Chem.
Soc..

[62] Brichtová E. P., Krupová M., Bouř P., Lindo V., Dos Santos A. G., Jackson S. E. (2023). Glucagon-like peptide 1 aggregates into low-molecular-weight
oligomers off-pathway to fibrillation. Biophys.
J..

[63] Poon S., Birkett N. R., Fowler S. B., Luisi B. F., Dobson C. M., Zurdo J. (2009). Amyloidogenicity and aggregate cytotoxicity of human glucagon-like
peptide-1 (hGLP-1). Protein Pept. Lett..

[64] Miller M. A., Wales D. J. (2005). Novel structural
motifs in clusters of dipolar spheres:
knots, links, and coils. J. Phys. Chem. B.

